# Regeneration of long-distance peripheral nerve defects after delayed reconstruction in healthy and diabetic rats is supported by immunomodulatory chitosan nerve guides

**DOI:** 10.1186/s12868-017-0374-z

**Published:** 2017-07-18

**Authors:** Lena Stenberg, Maria Stößel, Giulia Ronchi, Stefano Geuna, Yaobin Yin, Susanne Mommert, Lisa Mårtensson, Jennifer Metzen, Claudia Grothe, Lars B. Dahlin, Kirsten Haastert-Talini

**Affiliations:** 1 Department of Translational Medicine – Hand Surgery, Lund University, 20502 Malmö, Sweden; 2 Institute of Neuroanatomy and Cell Biology, Hannover Medical School, 30625 Hannover, Germany; 3 Department of Clinical and Biological Sciences, and Cavalieri Ottolenghi Neuroscience Institute, University of Turin, 10043 Orbassano, Turin Italy; 4 Division of Immunodermatology and Allergy Research, Department of Dermatology and Allergy, Hannover Medical School, 30625 Hannover, Germany; 5 Department of Hand Surgery, Skåne University Hospital, 20502 Malmö, Sweden; 6 Center for Systems Neuroscience (ZSN), 30559 Hannover, Germany

**Keywords:** Chitosan nerve guide, Delayed nerve reconstruction, Diabetes, Regenerative matrix, Immunomodulation

## Abstract

**Background:**

Delayed reconstruction of transection or laceration injuries of peripheral nerves is inflicted by a reduced regeneration capacity. Diabetic conditions, more frequently encountered in clinical practice, are known to further impair regeneration in peripheral nerves. Chitosan nerve guides (CNGs) have recently been introduced as a new generation of medical devices for immediate peripheral nerve reconstruction. Here, CNGs were used for 45 days delayed reconstruction of critical length 15 mm rat sciatic nerve defects in either healthy Wistar rats or diabetic Goto-Kakizaki rats; the latter resembling type 2 diabetes. In short and long-term investigations, we comprehensively analyzed the performance of one-chambered hollow CNGs (hCNGs) and two-chambered CNGs (CFeCNGs) in which a chitosan film has been longitudinally introduced. Additionally, we investigated in vitro the immunomodulatory effect provided by the chitosan film.

**Results:**

Both types of nerve guides, i.e. hCNGs and CFeCNGs, enabled moderate morphological and functional nerve regeneration after reconstruction that was delayed for 45 days. These positive findings were detectable in generally healthy as well as in diabetic Goto-Kakizaki rats (for the latter only in short-term studies). The regenerative outcome did not reach the degree as recently demonstrated after immediate reconstruction using hCNGs and CFeCNGs. CFeCNG-treatment, however, enabled tissue regrowth in all animals (hCNGs: only in 80% of animals). CFeCNGs did further support with an increased vascularization of the regenerated tissue and an enhanced regrowth of motor axons. One mechanism by which the CFeCNGs potentially support successful regeneration is an immunomodulatory effect induced by the chitosan film itself. Our in vitro results suggest that the pro-regenerative effect of chitosan is related to the differentiation of chitosan-adherent monocytes into pro-healing M2 macrophages.

**Conclusions:**

No considerable differences appear for the delayed nerve regeneration process related to healthy and diabetic conditions. Currently available chitosan nerve grafts do not support delayed nerve regeneration to the same extent as they do after immediate nerve reconstruction. The immunomodulatory characteristics of the biomaterial may, however, be crucial for their regeneration supportive effects.

**Electronic supplementary material:**

The online version of this article (doi:10.1186/s12868-017-0374-z) contains supplementary material, which is available to authorized users.

## Background

Transection or laceration injuries of peripheral nerves regularly need microsurgical reconstruction. This should be performed as early as possible, e.g. immediately after the trauma or within a few days [1]. Under certain circumstances, however, a delay of nerve reconstruction for up to several weeks is advisable. This can be the case after a motor-cycle accident resulting in brachial plexus lesions together with multiple other injuries, or after gun-shot or shrapnel widespread injuries where all injured tissue should have healed properly before nerve reconstruction and when the risk for infection is low [1]. The delay of microsurgical nerve reconstruction is, however, known for several reasons to result in worse axonal regeneration and a poor clinical outcome. Severe peripheral nerve lesions result in eventually life-long paralysis, severely impaired sensation, reduced functionality and/or pain [2]. The gold standard therapy for tension-free reconnecting nerve ends [3] is the use of autologous nerve grafts. Harvesting the nerve grafts is, however, accompanied by the need of a secondary surgery and loss of donor nerve function [4, 5]. Autologous nerve tissue additionally has a limited availability and brachial plexus lesions for example often need a considerably high amount of graft material. Therefore, alternatives to autologous nerve grafts are highly warranted [6].

Recently, we contributed to the development of a new generation of hollow chitosan nerve guides providing appropriate support of the regenerating rat sciatic nerve after reconstruction of 10–15 mm peripheral nerve gaps [7–9]. We additionally demonstrated that the degree of functional recovery after immediate reconstruction of 15 mm rat sciatic nerve gaps can even be increased by longitudinally introducing a perforated chitosan film into the hollow nerve guides as guidance structure for outgrowing axons [10].

The first objective of the current study relates to the growing incidence of a systemic diseases inflicting damage to the peripheral nervous system, i.e. diabetes. The global number of patients with type 1 and 2 diabetes will grow substantially in the coming decades. This is due to a real increase in incidence of type 1 diabetes as well as caused by an older population globally and thereby increasing the number of patients with type 2 diabetes, where the incidence is stable. Therefore, any type of new medical device should be tested in diabetic conditions; an aspect which is not often highlighted in the literature. This is of specific importance also for new nerve guides. Recently, we showed in short-term investigations in diabetic Goto-Kakizaki (GK) rats, with moderately increased and clinically relevant blood glucose levels, sufficient nerve regeneration through chitosan nerve guides and autologous nerve grafts bridging short and critical length sciatic nerve defects of up to 15 mm [10, 11]. Consequently, our first objective in the current study was to evaluate if sufficient nerve regeneration could also be observed in short-term evaluation in healthy Wistar and in diabetic GK rats after a delayed nerve reconstruction bridging the longer nerve defect (15 mm) with two different types of chitosan nerve guides that earlier have demonstrated their beneficial effect after acute nerve reconstruction [10]. Therefore, reconstruction of 15 mm rat sciatic nerve gaps has been delayed for 45 days. Reconstruction was performed using one-chambered hollow chitosan nerve guides (hCNGs) or two-chambered chitosan film enhanced chitosan nerve guides (CFeCNGs).

After general tissue regeneration, functional recovery after severe peripheral nerve injury and subsequent surgical reconstruction is highly important. Consequently, the second objective of the current study was to evaluate in long-term experiments if our chitosan nerve guides support functional recovery also after the surgical nerve reconstruction has been substantially delayed.

Finally, to make our conclusions round, we elucidated a potential mechanism by which the chitosan material used for our nerve guides could specifically support peripheral nerve regeneration. In the context of nerve graft reconstruction, an immunomodulatory effect on the polarization of invading macrophages towards a pro-healing phenotype has been described important for sufficient tissue regrowth and axonal regeneration [12]. Chitosan materials have demonstrated to induce similar immunomodulation, at least when implanted subcutaneously [13]. To prove that our chitosan material has indeed immunomodulatory and pro-healing properties for macrophages invading the nerve guides, we provide novel results from an in vitro evaluation of blood-derived human monocyte polarization with contact to our chitosan material.

## Results

### Experimental design

To induce a delayed nerve reconstruction scenario, the left sciatic nerve of each animal has been initially transected and spontaneous regeneration has been prevented by loop-like suturing the nerve ends to themselves as illustrated in Additional file 1: Figure S1.

After 45 days, the nerve reconstruction surgery was performed using either hCNGs or CFeCNGs to bridge a 15 mm rat sciatic nerve gap. Although grafting with autologous nerve tissue is the gold standard therapy in the clinical treatment of severe peripheral nerve injuries, this reconstruction approach could not be used as control here. At 45 days after injury, the nerve ends have considerably been pulling away from each other during the delay phase. This resulted in an unavailability of enough ipsilateral autologous sciatic nerve material to be used as the commonly applied reversed autograft known from immediate reconstruction studies [7, 10]. Since we had no ethical permission to harvest autologous nerve tissue from the contralateral side, which would also have inflicted the functional evaluation, and the use of additional donor animals was determined an inappropriate increase of animals to be used in this study, we focused this study on the evaluation of putative differences in the performances of CFeCNGs compared to hCNGs that are already clinically used. Table 1 provides an overview of the two parallel studies and the respective read-outs performed.

**Table 1:** Overview of the experimental design of the two parallel sub-studies

Study	Observation time (days)	Experimental groups	n	Group name	Performed experiments
Study I (ULUND)	56	Healthy rats: Hollow chitosan nerve guide	5	hCNG-I^healthy^	Analysis of regenerative matrix and distal nerve segment: Presence of axons and activated and apoptotic Schwann cells by immunohistology; Analysis of sensory dorsal root ganglion activation and neuroprotection by immunohistology
		Diabetic GK rats: Hollow chitosan nerve guide	11	hCNG-I^diabetic^	
		Healthy rats: Chitosan film enhanced chitosan nerve guide	6	CFeCNG-I^healthy^	
		Diabetic GK rats: Chitosan film enhanced chitosan nerve guide	7	CFeCNG-I^diabetic^	
Study II (MHH)	150	Hollow chitosan nerve guide	10	hCNG-II	Motor recovery: Non-invasive electrophysiology (60, 90, 120, 150 days) and muscle weight ratio; Histomorphometry; Immunohistology
		Chitosan film enhanced chitosan nerve guide	7	CFeCNG-II	

*GK* Goto-Kakizaki rats, *hCNG* hollow chitosan nerve guides, *CFeCNG* chitosan film enhanced chitosan nerve guides, *I* short-term experiments, *II* long-term experiments, *ULUND* University of Lund, *MHH* Medizinische Hochschule Hannover, i.e. Hannover Medical School

Study I (56 days short-term observation) was conducted in generally healthy or diabetic Goto-Kakizaki (GK) rats [14] and evaluated the following groups: hCNG-I^healthy^, hCNG-I^diabetic^, CFeCNG-I^healthy^, and CFeCNG-I^diabetic^ (Table 1). With immunohistological techniques, the formed regenerative matrix (if formed) and distal nerve segment (axonal outgrowth, Schwann cell activation and apoptosis) as well as the activation and neuroprotection of sensory dorsal root ganglion (DRG) neurons were analyzed [10].

Study II (150 days long-term observation) included the following groups: hCNG-II and CFeCNG-II. A mismatch in animal numbers in study I and II was created by the fact that some animals had to be excluded from the study before actual nerve reconstruction due to signs of automutilation. Functional recovery was periodically assessed and completed by endpoint histology and morphometrical analysis of axonal regeneration; the latter was executed at the Geuna lab at University of Turin (UNITO, Italy).

### Short-term evaluation of the regenerative matrix and dorsal root ganglia at 56 days after delayed nerve reconstruction (study I)

Preoperative blood glucose levels differed between the healthy Wistar rats and the diabetic GK rats (Table 2), but did not differ between the rats from the hCNG or CFeCNG groups. The blood glucose values in the corresponding groups did not differ pre- and postoperatively (results not shown).

**Table 2:** Nerve regeneration in healthy Wistar rats and in diabetic Goto-Kakizaki (GK) rats

	hCNG-I^healthy^ (n = 5)	CFeCNG-I^healthy^ (n = 6)	hCNG-I^diabetic^ (n = 11)	CFeCNG-I^diabetic^ (n = 7)	p-values (KW)	Fisher’s method^a^	
						hCNG-I/CFeCNG-I	Healthy/diabetic
Preoperative B-glucose (mmol/l)	3.9 [3.7–4.3]	4.0 [3.4–4.1]	7.9 [7.1–8.7]	8.1 [6.7–8.6]	*0.0001*	0.96	*<0.0001*
Regenerative Matrix Present (non-complete/complete single-strand/complete double-strand)	1/4/0	2/2/2	8/3/0	0/1/6	NA	NA	NA
Axonal outgrowth (neurofilament present in SND)	5/5	6/6	11/11	7/7	NA	NA	NA
ATF-3 (% of total)							
At 3 mm	8.1 [3.0–9.1]	8.8 [6.2–10.6]	6.0 [4.5–10.4]	9.5 [5.2–11.1]	0.51	NA	NA
In conduit	TFO	11.4 [6.8–12.7]^d^	16.8 [15.8–22.3]^b^	14.2 [10.3–22.5]	0.29	NA	NA
In SND	7.3 [6.2–16.7]	10.2 [8.6–12.9]	12.2 [9.4–14.3]	6.5 [6.0–11.1]	0.23	NA	NA
Cleaved caspase-3 (% of total)							
At 3 mm	30.5 [27.4–37.7]	31.2 [25.2–33.1]	31.9 [28.8–46.8]	19.1 [14.6–20.1]	*0.004*	*0.001*	*0.02*
In conduit	TFO	TFO	14.2 [12.5–16.1]^c^	12.9 [11.1–17.6]	0.082	NA	NA
In SND	28.0 [22.1–33.5]	28.6 [25.2–38.8]	37.8 [27.1–41.7]	37.4 [30.7–39.2]	0.36	NA	NA
DAPI stained cells (no/mm^2^)							
At 3 mm	167 [139–195]	156 [129–185]	184 [152–206]	169 [151–232]	0.59	NA	NA
In conduit	TFO	3490 [3362–3980]^d^	1727 [1412–2921]	3198 [1991–3635]	0.21	NA	NA
In SND	2823 [2201–3234]	2542 [2083–3036]	3071 [2445–3454]	2732 [2330–3733]	0.60	NA	NA

At 45 days post injury, a 15 mm long sciatic nerve defect has been bridged by a one-chambered hollow chitosan nerve guide (hCNG-I) or a two-chambered chitosan nerve guide with a centrally introduced chitosan film (CFeCNG-I). Evaluation was performed after a further 56 days. Values are median 25th(Q1)–75th(Q3) percentiles

Italic p-values indicate statistically significant differences (p < 0.05) between the examined groups

*KW* Kruskal–Wallis, *SND* distal nerve segment, *TFO* too few observations to apply statistics, *NA* not applicable (i.e. KW = Kruskal–Wallis not significant)

^a^Fisher’s method for independent samples based on the Chi square distribution from the subsequent Mann–Whitney test after Kruskal–Wallis test

^b^6 missing values

^c^7 missing values

^d^2 missing values

In the following, we describe the results of multiple immunohistological analyses. For all stainings performed, control procedures with omitting primary or secondary antibodies were performed and resulted in unstained sections (not presented).

The described procedures have been performed in different previous studies and are routine procedures to analyze the regenerating conditions short-term after peripheral nerve reconstruction with tubular grafts [7, 10, 11, 14].

### Regenerative matrix

The formation of a regenerative matrix is a prerequisite for successful regeneration of a peripheral nerve through a tubular graft [15]. A complete regenerative matrix, i.e. extending and completely connecting the proximal and distal nerve ends, was not detected macroscopically in all conduits. A complete single-strand matrix was observed in 4/5 hCNG-I^healthy^ rats, while a complete single-strand was only observed in 3/11 hCNG-I^diabetic^ rats. No complete separate strands were detected in the other rats of these two groups. In the six CFeCNG-I^healthy^ rats, a complete single-strand matrix was observed in two rats and a complete double-strand matrix in another two rats. In contrast, a complete double-strand matrix was observed in 6/7 CFeCNG-I^diabetic^ rats and a complete single-strand in the last CFeCNG-I^diabetic^ rat (Table 2).

The regenerative matrices were relatively thin and therefore not all of immunohistological procedures described in the following could be performed in all of them.

### Axonal outgrowth

Within the distal nerve segment, the presence of axons immunopositive for neurofilament indicates that regenerating axons have crossed the regenerative matrices within the nerve guides. Positive neurofilament staining was detected in the distal nerve segment in all the rats in all groups without any obvious differences between the groups (Table 2). The regenerative matrices within the conduits were extremely thin and therefore not additionally analyzed for the presence of neurofilament immunoreactivity.

Priority was given to analyses of activated and apoptotic Schwann cells in the formed matrices as described in the following.

### Activated Schwann cells

**Fig. 1:**
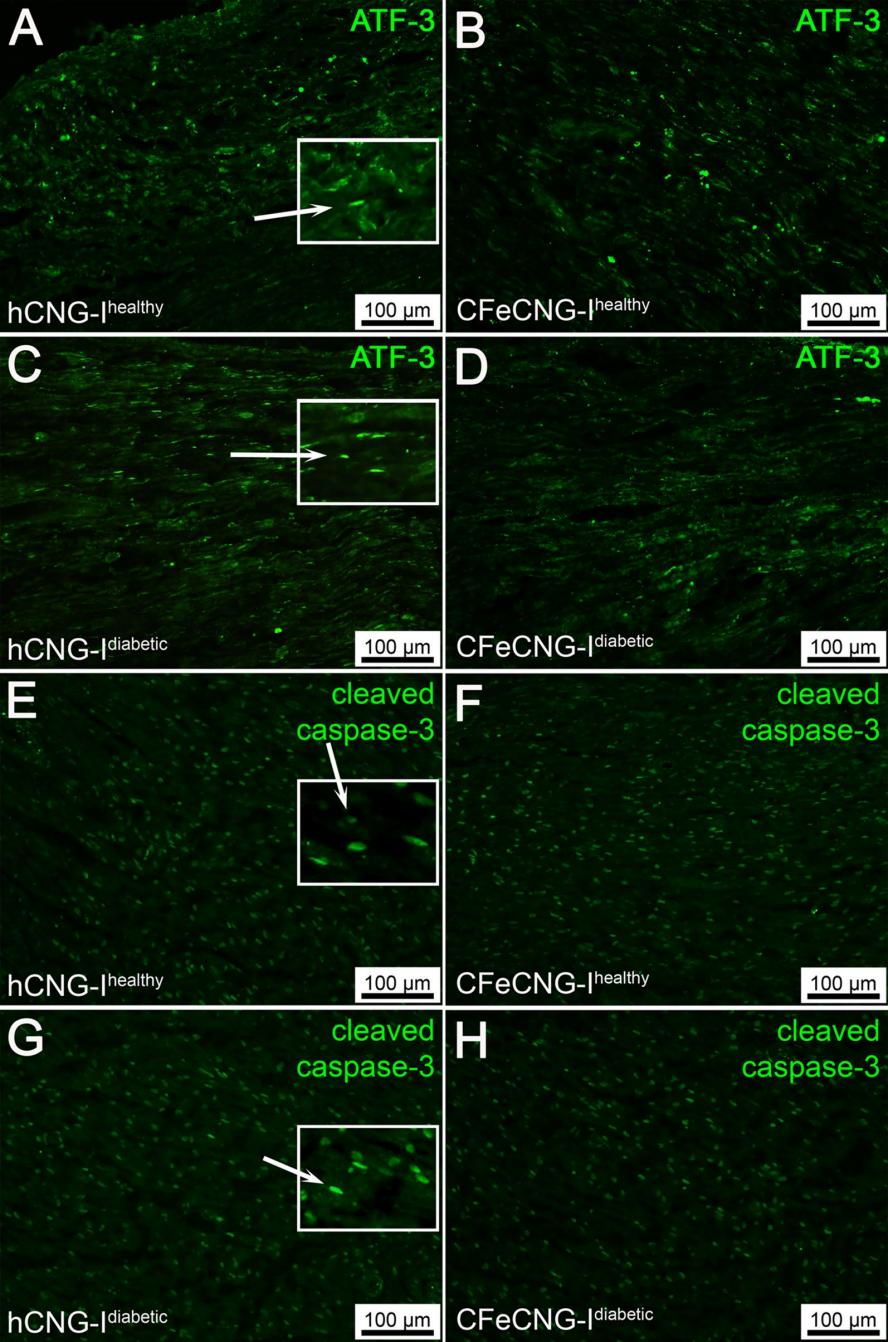
Distal nerve segments 56 days after delayed nerve reconstruction stained for ATF-3 and cleaved caspase-3. **A**–**D** Photomicrographs present immunohistology for ATF-3. **E**–**H** Photomicrographs present immunohistology for cleaved caspase-3. *Inserts* are magnifications from the image showing the oval ATF-3 (rabbit anti-ATF-3 polyclonal antibody) and cleaved caspase-3 (anti-cleaved caspase-3 antibody) stained cells interpreted as Schwann cells (secondary antibody Alexa Fluor 488 conjugated goat anti-rabbit IgG; green; see methods and Additional file 2: Figure 2). *hCNG-I* ^*healthy*^ hollow chitosan nerve guide from healthy rats, *CFeCNG-I* ^*healthy*^ chitosan film enhanced chitosan nerve guide from healthy rats, *hCNG-I* ^*diabetic*^ hollow chitosan nerve guide from diabetic GK rats, *CFeCNG-I* ^*diabetic*^ chitosan film enhanced chitosan nerve guide from diabetic rats. For details of the results of the quantification see Table 2. *Scale bars* display 100 µm in all images

Activated Schwann cells, defined as ATF-3 stained Schwann cells [10, 11, 14], were observed in the formed regenerative matrix in generally small proportions (median values 6.0–16.8%). The values displayed no significant differences between the groups (Table 2; it should be recognized that only few matrices were available for immunostaining in the middle of the conduit, particularly in the hCNG-I^healthy^ samples). Furthermore, the proportion of ATF-3 stained Schwann cells in the distal nerve segment (Fig. 1A–D) did not significantly differ (median values 6.5–12.2%, Table 2). No differences were found between healthy and diabetic GK rats. Double-staining of ATF-3 and S-100 (Additional file 2: Figure S2A–D) was done to show that the ATF-3 staining was located in the oval cell nuclei of S-100 immunopositive Schwann cells in accordance with previous studies [11].

### Apoptotic Schwann cells

Staining for cleaved caspase-3 was used to detect apoptotic Schwann cells in the regenerative matrix and in the distal nerve segment [10, 11, 14]. The proportions of cleaved caspase-3 immunopositive Schwann cells differed between the groups at 3 mm distal to the proximal suture in the formed matrices, where the Fisher test indicated a lower percentage in the diabetic GK rats versus healthy Wistar rats and in the CFeCNG-I samples versus hCNG-I samples (Table 2). No statistical differences, based on the possibility to apply statistics, were observed in the middle of the conduit (but again, it should be recognized that only few matrices were available for immunostaining in the middle of the conduit, particularly in the hCNG-I^healthy^ samples and that the results should be interpreted with caution) or in the distal nerve segment (Fig. 1E–H). Again, no differences were found between healthy Wistar rats and diabetic GK rats. Double-staining of cleaved caspase-3 and S-100 was done to show that the cleaved caspase-3 staining was located in the oval cell nuclei of S-100 immunopositive Schwann cells in accordance with previous studies (Additional file 2: Figure S2E–H, [10]).

### Total number of cells

Nuclei of all cells were counterstained with DAPI in order to define the percentage of activated or apoptotic Schwann cells in the analyzed areas. There were no significant differences between the groups concerning the total number of DAPI stained cells in the matrix at 3 mm from the proximal suture, in the matrix in the middle of the conduit and in the distal nerve segment (Table 2). However, only few matrices were possible to evaluate in the hCNG-I^healthy^ rats and statistics could not be applied to that group.

### Dorsal root ganglia

Sensory dorsal root ganglia located at the lumbar level (L3-L5) should regenerate their peripheral axons into the reconstructed nerve for recovery of e.g. nociception, mechanosensitivity and proprioception. In order to do so, they need to be protected from axotomy-induced cell death. Evaluation of ATF-3 and HSP-27 immunoreactivity of the corresponding dorsal root sensory neurons revealed their regeneration state and the results are described in the following.

### Activated sensory neurons

**Fig. 2:**
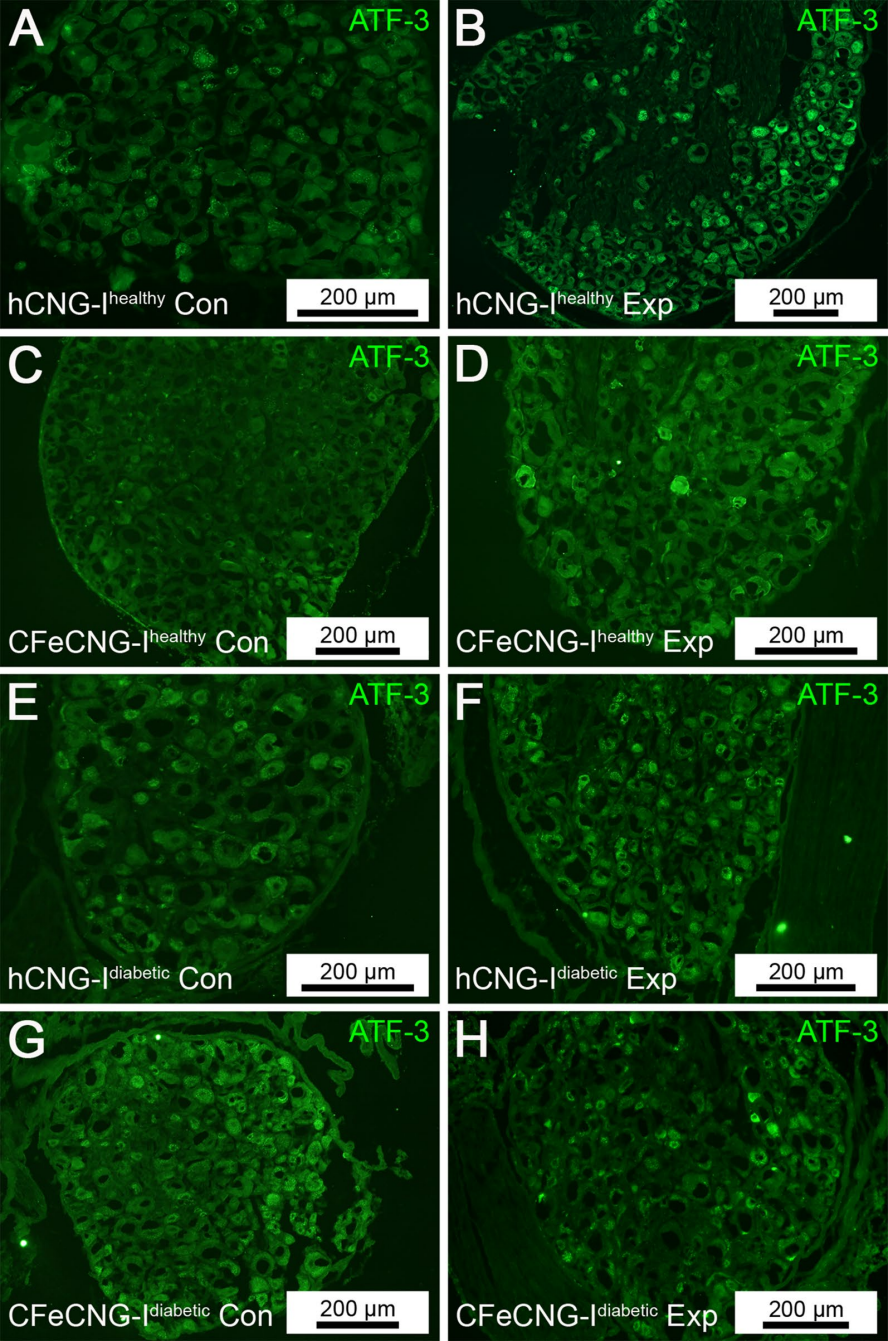
Dorsal root ganglia at 56 days after delayed nerve reconstruction stained for ATF-3. Photomicrographs present ATF-3 stained sensory dorsal root ganglion neurons (*green*) from the uninjured control side (Con, *left column*; **A**, **C**, **E**, **G**) and the experimental side (Exp, *right column*; **B**, **D**, **F**, **H**). *hCNG-I* ^*healthy*^ hollow chitosan nerve guide from healthy rats, *CFeCNG-I* ^*healthy*^ chitosan film enhanced chitosan nerve guide from healthy rats, *hCNG-I* ^*diabetic*^ hollow chitosan nerve guide from diabetic GK rats; *CFeCNG-I* ^*diabetic*^ chitosan film enhanced chitosan nerve guide from diabetic rats. For details of the results of the quantification see Table 3. *Scale bars* display 200 µm in all images (*scale bars* represent different lengths due to different sizes of the DRG sections)

Activated sensory neurons, i.e. ATF-3 stained neurons (Fig. 2), were evaluated in the sensory neurons in DRGs according to a previously published method [10]. No differences were found on the control side, which revealed significantly smaller proportions of ATF-3 immunopositive neurons than on the experimental side (Table 3). On the experimental side, generally a small proportion of ATF-3 stained sensory neurons were detected (5.0–5.7%). Significantly bigger proportions, however, were observed in the DRGs from CFeCNG-I healthy or diabetic rats when compared to DRGs from hCNG-I healthy or diabetic rats (Table 3). No statistical differences were found between healthy and diabetic rats.

**Table 3:** Immunohistology for activated and neuroprotected sensory neurons in healthy and diabetic rats after nerve reconstruction

Treatment	Immunohistology of dorsal root ganglia (DRG; L4/L5)				
	ATF-3 control (% of total sensory neurons)	ATF-3 experimental (% of total sensory neurons)	HSP-27 control (% of area in sections of DRG)	HSP-27 experimental (% of area in sections of DRG)	HSP-27 ratio Exp/Con
hCNG-I^healthy^ (n = 5)	1.4 [1.0–2.9]	3.1 [2.9–4.0]^c^	9.9 [7.6–10.1]	16.5 [15.6–18.5]^g^	1.9 [1.7–2.1]
hCNG-I^diabetic^ (n = 11)^k^	1.8 [1.1–2.6]	4.6 [3.7–4.9]^d^	6.6 [6.0–6.9]	17.1 [15.3–19.8]^h^	2.6 [2.4–3.0]
CFeCNG-I^healthy^ (n = 6)^k^	1.5 [1.2–1.8]	5.0 [4.9–6.1]^e^	8.2 [6.5–10.6]	16.4 [11.0–23.5]^i^	1.7 [1.5–2.7]
CFeCNG-I^diabetic^ (n = 7)	2.3 [1.2–2.6]	5.7 [5.0–6.0]^f^	8.3 [5.4–9.1]	18.2 [14.9–19.0]^j^	2.2 [2.1–2.8]
p-value^a^ (KW)	0.77	*0.001*	0.06	0.84	*0.034*
Fisher’s method^b^					
hCNG-I/CFeCNG-I	NA	*0.0001*	NA	NA	0.36
Healthy/diabetic	NA	0.14	NA	NA	*0.004*

Activation was detected by ATF-3 immunostaining and neuroprotection by HSP-27 immunostaining in dorsal root ganglion sensory neurons at 56 days after delayed nerve reconstruction in healthy Wistar rats and diabetic Goto-Kakizaki (GK) rats. At 45 days post injury a 15 mm long sciatic nerve defect was bridged by a hollow chitosan nerve guide (hCNG-I) or a chitosan nerve guide with a centrally introduced chitosan film (CFeCNG-I)

Values are median (interquartile range; IQR)

Italic p-values indicate statistically significant differences (p < 0.05) between the examined groups

NA = not applicable (i.e. KW = Kruskal–Wallis not significant)

^a^KW = Kruskal–Wallis

^b^Fisher’s method for independent samples based on the Chi square distribution

^c–j^Wilcoxon = statistical difference between experimental and control sides; ^c^ 0.043; ^d^ 0.005; ^e^ 0.043; ^f^ 0.018; ^g^ 0.043; ^h^ 0.005; ^i^ 0.043; ^j^ 0.018; respectively

^k^One missing value in the group

### Area of HSP-27 stained sensory neurons

**Fig. 3:**
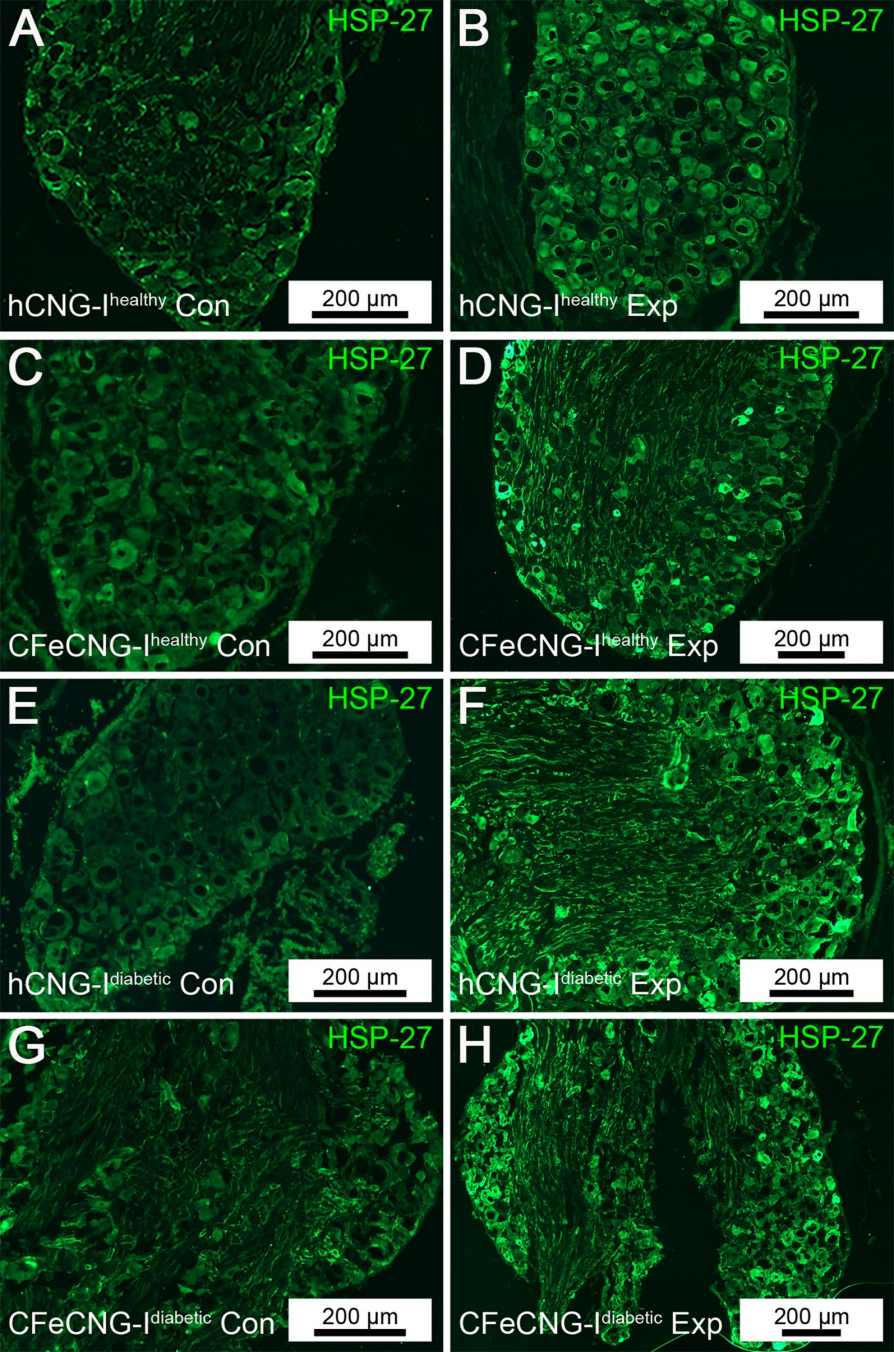
Dorsal root ganglia at 56 days after delayed nerve reconstruction stained for HSP-27. Photomicrographs present HSP-27 stained sensory dorsal root ganglion neurons (*green*) from the uninjured control side (Con, *left column*; **A**, **C**, **E**, **G**) and the experimental side (Exp, *right column*; **B**, **D**, **F**, **H**). *hCNG-I* ^*healthy*^ hollow chitosan nerve guide from healthy rats, *CFeCNG-I* ^*healthy*^ chitosan film enhanced chitosan nerve guide from healthy rats, *hCNG-I* ^*diabetic*^ hollow chitosan nerve guide from diabetic GK rats, *CFeCNG-I* ^*diabetic*^ chitosan film enhanced chitosan nerve guide from diabetic rats. For details of the results of the quantification see Table 3. *Scale bars* display 200 µm in all images (*bars* represent different lengths due to different sizes of the DRG sections)

The expression of the neuroprotective protein HSP-27 was evaluated in sensory neurons (Fig. 3) in DRGs according to a previously described method, being different from the assessment of ATF-3 stained sensory neurons (see above) [10]. On the control side, the HSP-27 expressing area in the sections from DRGs was 6.6–9.9% of the complete area of the section with no difference between the groups (Table 3). On the contralateral experimental side, 16.4–18.2% of the DRG areas were HSP-27 immunopositive, again with no differences between the groups. However, if a ratio between the HSP-27 expressed area on the experimental and control sides was calculated [10], a statistical difference was observed with higher values in the diabetic GK rats (Table 3), indicating a higher degree of neuroprotective mechanisms acting in diabetes after the present nerve injury and reconstruction. Interestingly, higher HSP-27 levels in serum are generally associated with better nerve function in both healthy subjects and in patients with type 2 diabetes although lower levels are found in type 2 patients [16].

### Correlations for all rats examined

Relevant correlations were also analyzed when the results from all rats were pooled together, but not in individual groups due to at times only few observations in each group. The preoperative blood glucose values correlated with the proportions of cleaved caspase-3 immunopositive Schwann cells (hCNG-I and CFeCNG-I pooled) in the distal nerve segment (r = 0.426, p = 0.02). The preoperative blood glucose levels also correlated negatively with the HSP-27 on the control side (r = −0.55, p = 0.003), but positively with the HSP-27 ratio (r = 0.50, p = 0.008).

The proportions of cleaved caspase-3 immunopositive apoptotic Schwann cells in the regenerative matrix at 3 mm from the proximal suture correlated negatively with the proportions of ATF-3 stained sensory neurons in DRGs on the experimental side (r = −0.41, p = 0.034). The proportions of cleaved caspase-3 immunopositive apoptotic Schwann cells in the distal nerve segment correlated negatively with the HSP-27 immunopositive area on the control side (r = −0.42, p = 0.03).

The HSP-27 ratio positively correlated with the HSP-27 immunopositive area on the experimental side (r = 0.51, p = 0.007). Furthermore, the HSP-27 ratio also correlated negatively with the HSP-27 expressing area on the control side (r = −0.59, p = 0.001).

### Assessment of functional motor recovery over a period of 150 days after delayed nerve reconstruction (study II)

The first measure to detect successful reinnervation of motor targets after delayed reconstruction of a 15 mm sciatic nerve gap in rats was electrodiagnostic recording of evoked compound muscle action potentials (CMAPs) from a more proximal lower limb target muscle (anterior tibial muscle) and the plantar interosseous muscles located more distal in the paw of the animals. Table 4 summarizes the timeline of the reinnervation processes as determined in periodic measurements.

**Table 4:** Timing for recovery of evocable CMAPs

Muscle	Nerve guide	60 days		90 days		120 days		150 days	
		Animals/group	[%]	Animals/group	[%]	Animals/group	[%]	Animals/group	[%]
*Tibialis anterior*	hCNG-II	1/10	10*	4/10	40	5/10	50	5/10	50
	CFeCNG-II	0/7	0	1/7	14	3/7	43	5/7	71*
*Plantar interosseus*	hCNG-II	0/10	0	0/10	0	1/10	10*	1/10	10*
	CFeCNG-II	0/7	0	0/7	0	0/7	0	0/7	0

The table gives a timeline of functional motor recovery detected as evocable CMAPs recorded from the tibialis anterior or plantar muscles upon transcutaneous stimulation of the reconstructed sciatic nerve. * Indicates a significant difference (p < 0.05) between hCNG-II and CFeCNG-II at the same time point and for the same muscle as determined in the Chi square test

Analyzing the qualitative parameter of functional muscle reinnervation (*yes* or *no*), a positive response was detectable earlier in the hCNG-II group than in the CFeCNG-II group. At 60 and 90 days post-surgery, regenerating axons in the CFeCNG-II group displayed a lower proportion of reinnervated *tibialis anterior* muscles, while a considerable higher percentage (10 and 40%, respectively) of animals in the hCNG-II group demonstrated reinnervation of these lower limb muscles. From 120 days after surgery onwards, the CFeCNG-II group caught up and demonstrated a comparable percentage of reinnervated *tibialis anterior* muscles (43 vs. 50% in hCNG-II group) with even surpassing the percentage in the hCNG-II group (stabilized 50% vs. further elevated 71% in CFeCNG-II group) at day 150. Reinnervation of the *plantar interosseous* muscle, however, did occur exclusively in a single animal of the hCNG-II and was detectable from 120 days post-surgery onward.

Quantitative parameters, like the CMAP amplitude ratios and the nerve conduction velocities ratios recorded from the tibialis anterior muscles, were additionally analyzed and are depicted in Additional file 3: Figure 3A and B. It is clearly visible from the dot plots that among the animals that displayed electrodiagnostically detectable reinnervation, no significant difference occurred between the two groups. The muscle weight ratio (Additional file 3: Figure S3C) was calculated as a second measure to quantify the extent of muscle reinnervation that was in accordance with the previous result.

### Macroscopic inspection at the time of explantation (150 days after delayed nerve reconstruction)

Just before tissue harvest for histological processing, a careful macroscopic observation of the reconstructed nerves in study II at 150 days after the delayed reconstruction surgery was performed. As summarized in Table 5, we detected a discrepancy between the visible tissue regeneration and the electrodiagnostically detectable functionality of the nerves.

**Table 5:** Correlation of electrodiagnostically detectable motor recovery with macroscopic tissue regeneration

Nerve guide	n	Evocable CMAPs (tibialis anterior muscle)		Regenerated tissue without evocable CMAP		No regenerated tissue	
		Animals/group	[%]	Animals/group	[%]	Animals/group	[%]
hCNG-II	10	5	50	3	30	2	20
CFeCNG-II	7	5	71*	2	29	0	0*

The table illustrates the relationship between electrodiagnostically detectable reinnervation of the tibialis anterior muscles and the visibility of a regenerated tissue bridge between the previous nerve ends within the nerve guides. In both groups, more tissue bridges have been regenerated than axonal outgrowth had already established reinnervation of the evaluated target muscles at 150 days after delayed nerve reconstruction. In the hCNG-II, however, also a complete failure of regeneration occurred in 2 animals. * Indicates a significant difference (p < 0.05) between hCNG-II and CFeCNG-II (Chi square test)

While in two animals of the hCNG-II group a complete failure of regeneration occurred, we detected in 3/10 animals a tissue bridge between the former nerve ends additionally to the tissue bridges found in 5/10 animals with evocable CMAPs within the tibialis anterior muscle upon transcutaneous sciatic nerve stimulation directly proximal and distal to the nerve guide. In the CFeCNG-II group a more favorable balance was detected as all animals demonstrated tissue bridges connecting the former nerve ends within the nerve guides and the percentage of animals without detectable CMAPs was similar to that in the other group.

**Fig. 4:**
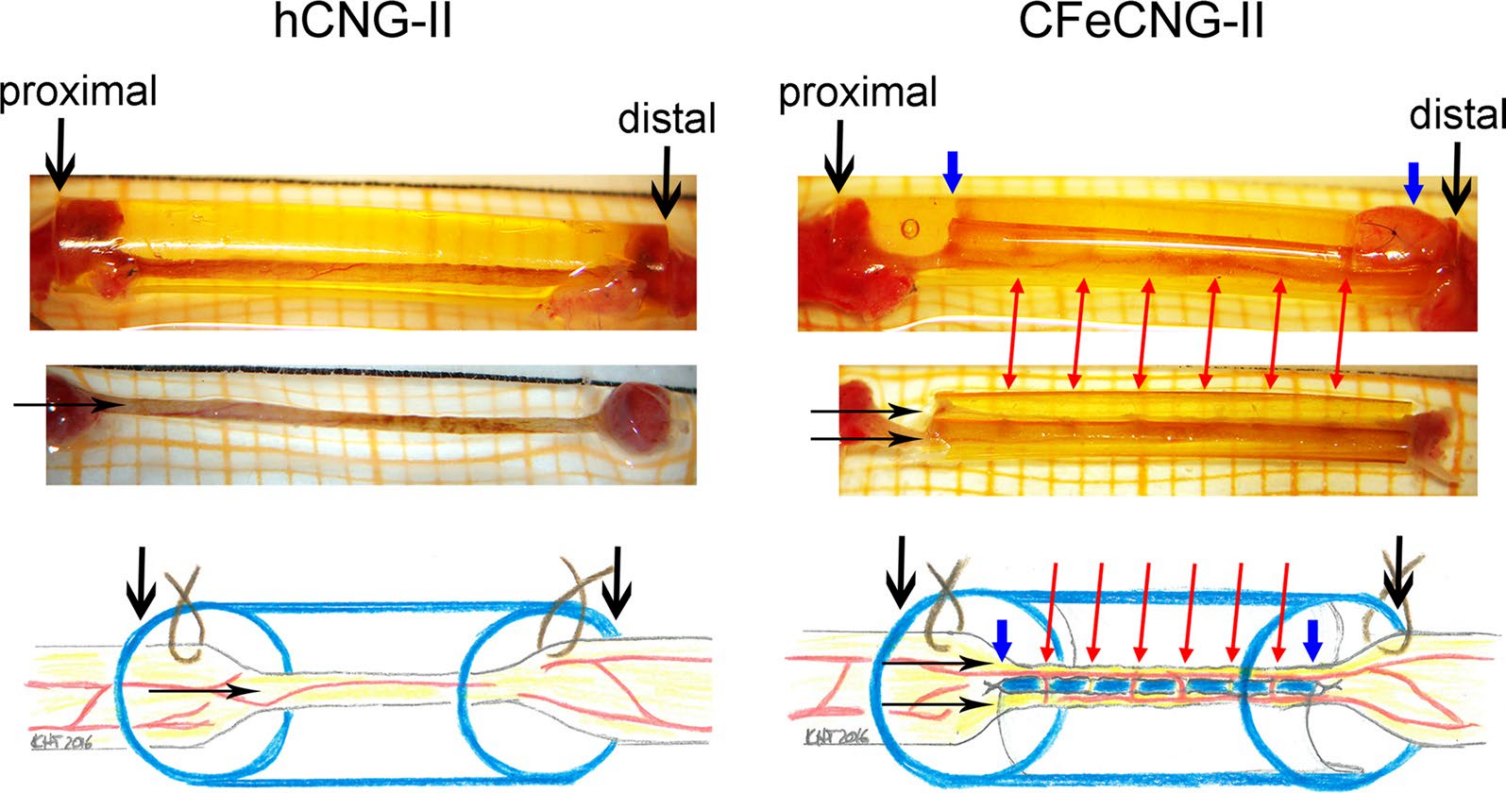
Presentation of the macroscopic appearance of the regenerated tissue upon explantation. At 150 days after delayed nerve reconstruction, the implants were explanted. Photographs and schematic drawings illustrate the macroscopic appearance of the regenerated tissue. *Top row* Representative photographs of regenerated tissue within the nerve guides (*left* hCNG-group, *right* CFeCNG-group). *Center row* Representative photographs of regenerated tissue after removal of the nerve guide (*left* hCNG-group, *right* CFeCNG-group with chitosan film still in place). *Bottom row* Schematic drawing of the regenerated tissue within the nerve guides (*left* hCNG-group, *right* CFeCNG-group), chitosan material is shown in *blue*. (1) *Downwards-pointing in black* indicating the outer edge of the chitosan nerve guide; (2) *downwards-pointing in blue* indicating the proximal and distal edge of the central chitosan film; (3) *horizontal arrows in black* indicating either single-stranded (hCNG-II) or double-stranded (CFeCNG-II) regenerated tissue; (4) *red arrows* point towards eventually vascularized tissue bridges between the two tissue cables in CFeCNGs

With the help of a surgical microscope it was possible to visualize that in one-chambered hollow hCNGs the regenerated tissue bridges were single-stranded, whereas in the two-chambered CFeCNGs they appeared double-stranded. As illustrated in Fig. 4, the latter were connected to each other by small tissue bridges grown through the holes within the central chitosan films. Additionally, these connections eventually contained small vessels resulting in a visually increased vascularization of the regenerated tissue within the CFeCNGs.

### Histomorphometrical evaluation at 150 days after delayed nerve reconstruction (study II)

#### Nerve histochemistry and immunohistology

**Fig. 5:**
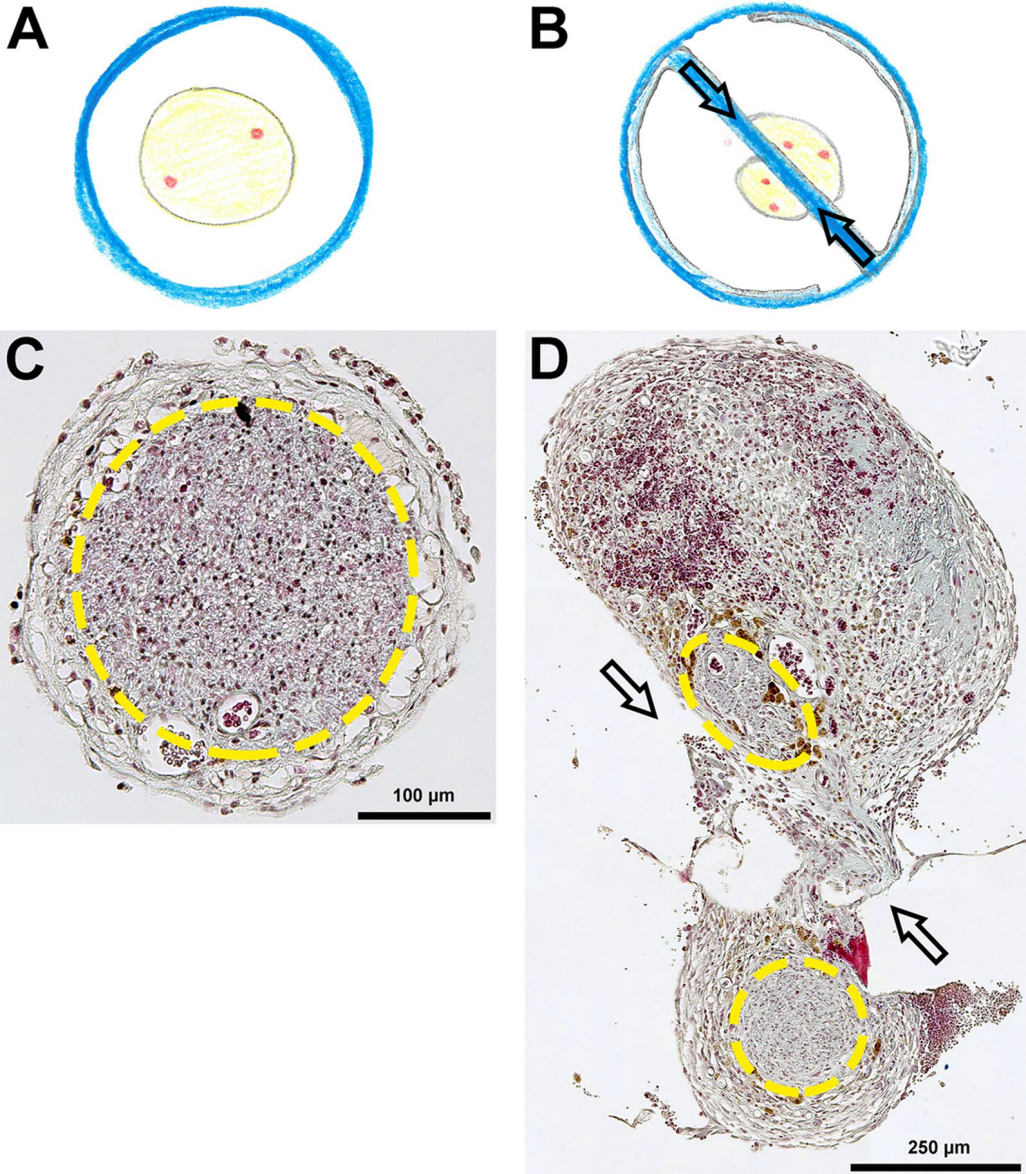
Presentation of the morphological appearance of the regenerated tissue within the nerve guides. Schematic drawings and photomicrographs illustrate the morphology of the regenerates formed within the different nerve guides during 150 days after delayed reconstruction. **A**, **B** Schematic drawing of the regenerated tissue within the nerve guides, chitosan material is shown in *blue* (**A** hCNG-II group, **B** CFeCNG-II group). **C**, **D** Representative photomicrographs of trichrome-stained cross-sections through the regenerated tissue at distal location within the nerve guides (**C** hCNG-II group, **D** CFeCNG-II group). *Yellow lines* encircle areas of axonal regeneration as visualized in immunohistology (Fig. 6). *Arrows* indicate the location of the central chitosan film (**B**, **D**) and a tissue bridge that has formed through one of the holes in it connecting the two strands of the regenerate (**D**)

Additionally to the macroscopic inspection, we performed histology of the regenerated tissue in order to demonstrate the different organization of the regrowing axons within the different types of nerve guides. As shown in Fig. 5 cross-sections through the distal part of the regenerated tissue within the nerve guides revealed the single-stranded morphology of it within the hCNG-II nerve guides and the double-stranded one within the CFeCNG-II nerve guides. The double-stranded regenerates were commonly divided in one strand of bigger and one strand of smaller diameter, both containing regenerating axons in close vicinity to the chitosan film (Fig. 5). The trichrome staining further visualized that within the CFeCNG-II nerve guides a larger amount of connective tissue was formed, but that it did not contain any considerable amount of fibrotic collagen fibers which would have been demonstrated in clear green color (Fig. 5).

**Fig. 6:**
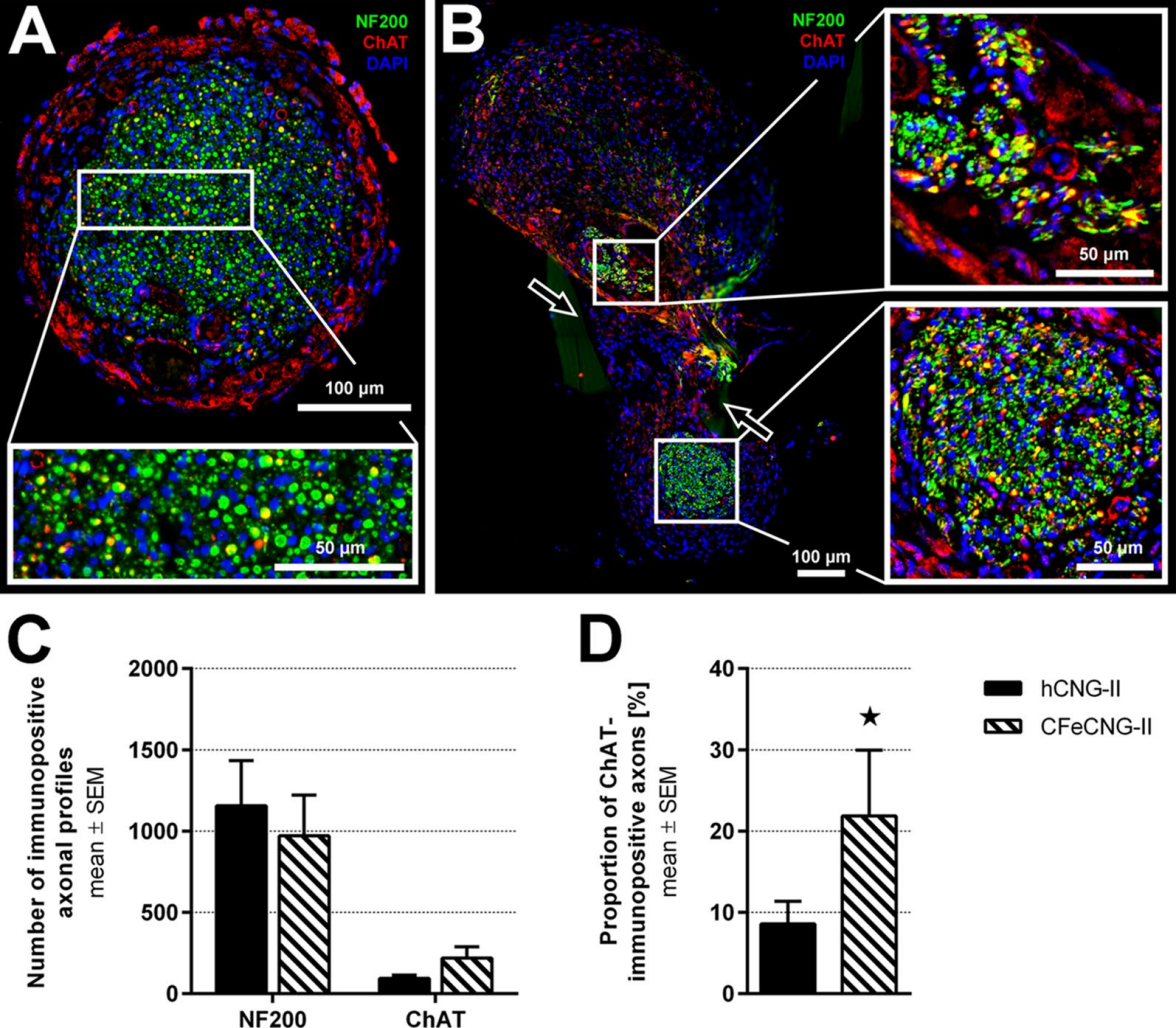
Immunohistology of cross-sections through distal parts of regenerated tissue within the nerve guides. At 150 days after delayed nerve reconstruction, immunohistological analysis of cross-sections through distal parts of the regenerated tissue within the nerve guides was performed. **A**, **B** Representative photomicrographs displaying the immunodetection of all regenerated axons (NF200 in *green*), regenerated motor axons (ChAT in *red*; co-localization with NF200 is indicated in *yellow*) and nuclear staining in *blue* (DAPI). *Arrows* in (**B**) indicate the location of the central chitosan film and a tissue bridge that has formed through one of the holes in it connecting the two strands of the regenerate. **C** Quantification of axonal profiles revealed a tendency to increased numbers of motor axons in the CFeCNG-II group. **D** The percentage of ChAT-immunopositive motor axons is significantly increased in CFeCNG-II samples (*p < 0.05). *Error bars* indicate mean ± SEM

Sections consecutive to those processed in trichrome staining were incubated with antibodies against the general axonal marker neurofilament 200 (NF200) and the marker for motor axons choline acetyl transferase (ChAT) to indicate the areas of axonal regeneration (Fig. 6A, B). Double-staining against NF200 and ChAT was further used to quantify the number of immunopositive axons (Fig. 6C) and to calculate the percentage of ChAT-immunopositive axons among all regenerated NF200-immunopositive axons (Fig. 6D). The percentage of regenerated motor axons (ChAT-immunopositive) was significantly increased in CFeCNG-II regenerates in comparison to hCNG-II regenerates.

#### Morphometric analysis

**Fig. 7:**
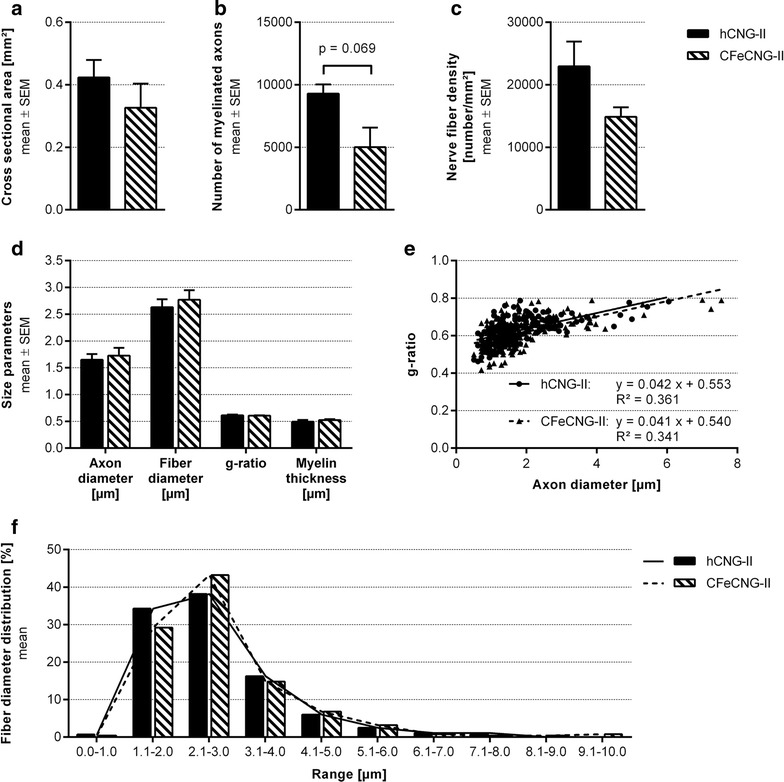
Nerve morphometry results of cross-sections through distal nerve segments. At 150 days after delayed nerve reconstruction, morpho-quantitative analysis of nerve segments distal to the nerve guides was performed. **a**–**d** Histograms depicting the cross-sectional area of the whole nerve section (**a**), the total number of myelinated fibers (**b**), the myelinated fiber density (**c**), and size parameters (axon and fiber diameter, g-ratio and myelin thickness) (**d**). Values are expressed as mean ± SEM. **e** *Scatter plots* showing g-ratios of individual myelinated axons as a function of axon diameter (obtained by pooling all values hCNG-II: n = 283 analyzed axons; CFeCNG-II: n = 250 analyzed axons). The *lines* represent linear regression lines. **f** Frequency distribution histograms of myelinated fiber diameters (obtained by pooling all values; hCNG-II: n = 283 analyzed axons; CFeCNG-II: n = 250 analyzed axons). The two lines are the fitting lines of the distribution

Three nerve segments of each group, harvested directly distal to the nerve guides (representative samples selected from animals with electrodiagnostically detectable motor recovery, Additional file 4: Figure S4), were subjected to stereological and morpho-quantitative analysis on semi-thin and ultra-thin cross-sections. As depicted in Fig. 7, the cross-sectional area was increased in the hCNG-II group (Fig. 7a), as was the total number of myelinated fibers (Fig. 7b), and the fiber density (Fig. 7c). But the differences were not significantly different between the hCNG-II and CFeCNG-II groups. Also size parameters (axon and fiber diameter, myelin thickness and g-ratio) were not different between the two experimental groups (Fig. 7d). Scatter plots displaying g-ratios of individual fibers in relation to their respective axon diameter also show no differences between groups and regression line equations (hCNG-II: y = 0.0421 x + 0.5525; CFeCNG-II: y = 0.0408 x + 0.5395) with comparable slope and intercept (Fig. 7f). When performing the frequency distribution of nerve fiber diameters, by pooling all values from the analyzed samples, both distributions were unimodal, but axons that have been re-growing along the central chitosan film in CFeCNG-II nerve guides appeared with slightly increased axonal diameters (histograms of fiber diameter are shifted to the right for CFeCNG-II samples in comparison to hCNG-II samples, Fig. 7e).

#### In vitro differentiation of human monocyte derived macrophages cultured on chitosan films

To elucidate if the process of macrophage differentiation contributes to the attenuated regeneration of peripheral axons along CFeCNGs after immediate [10] and also after delayed (current study) nerve reconstruction, we examined the differentiation behavior of monocyte derived macrophages in response to the chitosan films. Therefore, monocytes were isolated from human peripheral blood mononuclear cells obtained from buffy coats by adherence. The experiment was designed under strong consideration of translation aspects, because the basic hCNGs have already been approved for clinical use (Europe and USA, Reaxon^®^ Nerve Guide) and were used in more than 100 human patients since market entry two years ago [10]. Consequently, we found it more appropriate to investigate the chitosan effect on human peripheral blood-derived monocytes than on the same cells isolated from rat peripheral blood. Furthermore, chitosan effects on rat monocytes have already been described by others as will be discussed below.

Comparing the composition of the cultures plated onto regular cell culture surfaces (non-coated bottom of 24-well cell culture plate, regular well ground, WG) or chitosan films with a degree of ~5% acetylation (CF) under different culture conditions revealed that the CFs used in our studies provided an immunomodulatory effect and stimulated the immediate differentiation of macrophages towards the reparative M2 phenotype.

**Fig. 8:**
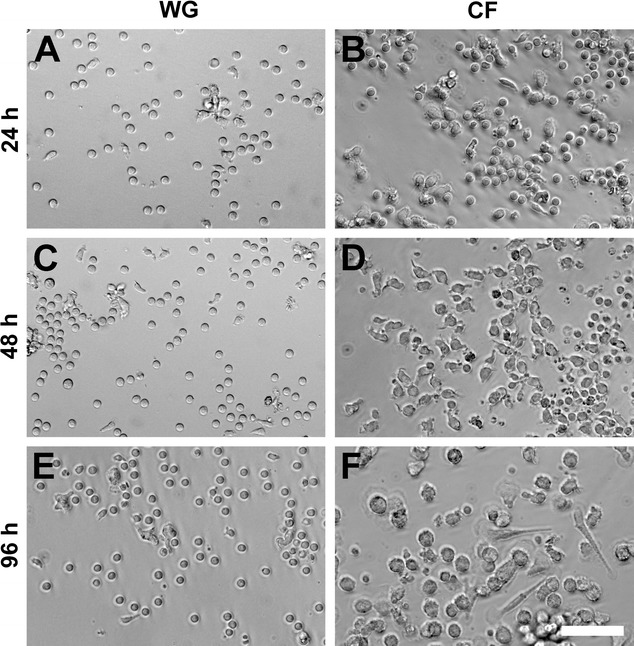
Morphology of macrophages cultivated on regular well grounds (WGs) or chitosan films (CFs). Representative photomicrographs on the morphological changes of macrophages cultivated either on WG (**A**, **C**, **E**) or CF (**B**, **D**, **F**). Displayed pictures indicate a strong time-dependency [e.g. at 24 h (**A**, **B**), 48 h (**C**, **D**), and 96 h (**E**, **F**)] of morphological changes of the macrophages cultivated on CFs. *Scale bar* represents 50 µm for all pictures

The differentiation towards the pro-regenerative M2 phenotype is initiated by the extension of pseudopodia from the originally round cells. This goes along with cell elongation and the subsequent upregulation of specific cell surface markers [17]. As illustrated in Fig. 8, cultivation of human blood-derived monocytes on CFs triggered a change in cell morphology. Already 24 h after seeding, first elongating cells were detectable on CF in cultures without other external stimuli (non-stimulated condition, Fig. 8B). The cells increased their flattened area with time and extend pseudopodia (Fig. 8D, F) in contrast to cells grown under the same non-stimulated conditions on regular well grounds (WG, Fig. 8C, E). When the culture medium was, however, supplemented with M-CSF, a cytokine that is known to promote differentiation of M2 macrophage phenotypes, also cells grown on WG developed pseudopodia (photomicrographs not shown).

Supplementation of M-CSF was used to simulate the immunomodulatory tissue conditions reported for peripheral nerves after injury, namely the anti-inflammatory stimulus that induces differentiation of monocytes towards the pro-healing M2 macrophage phenotypes [18].

**Fig. 9:**
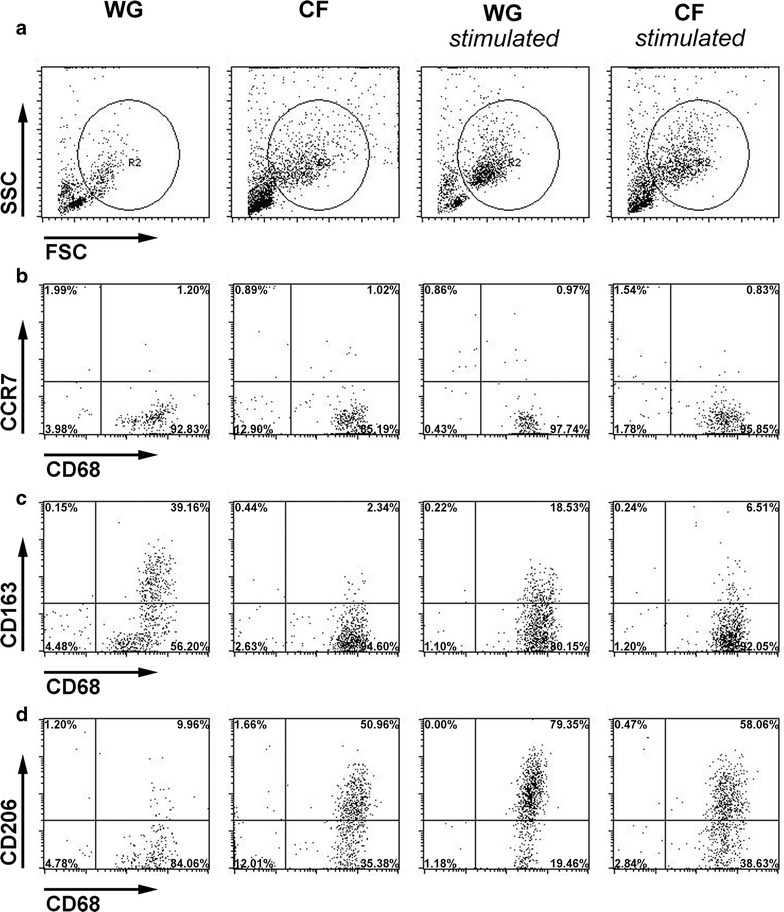
Cell surface marker expression profiles of non-stimulated or M-CSF-stimulated macrophages differentiated on CFs or WGs. Macrophages, cultivated for up to 2 days under different conditions, were harvested and stained with CD68- (macrophage differentiation marker independent of M1/M2 phenotype), CCR7- (M1 phenotype), CD163- (M2a phenotype) and CD206-antibodies (M2a and M2c phenotype) followed by FACS analysis. **a** The gate was set on the FSC versus SSC to analyze monocyte-derived cells and exclude dead cells and cell debris. **b** Surface expression of CCR7 and intracellular expression of CD68. **c** Surface expression of CD163 and intracellular expression of CD68. **d** Surface expression of CD206 and intracellular expression of CD68 *FSC* forward scatter, *SSC* side scatter, *WG* well ground, *CF* chitosan film (n = 4 experiments)

**Fig. 10:**
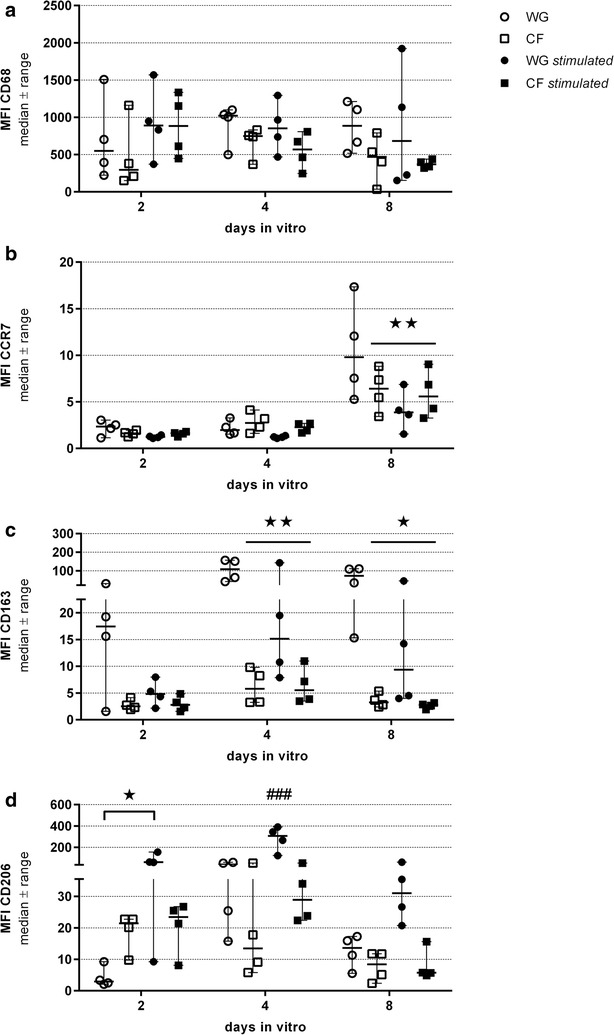
Characterization of cell surface markers of non-stimulated or M-CSF-stimulated macrophages differentiated on CFs or WGs. Macrophages were differentiated for 2, 4, and 8 days in vitro under different conditions, harvested and stained with CD68- (macrophage differentiation marker independent of M1/M2 phenotype), CCR7- (M1 phenotype), CD163- (M2a phenotype) and CD206-antibodies (M2a and M2c phenotype) before FACS analysis. Graphs represent the geometric mean fluorescence intensities (MFI) values of FSC versus SSC or CD68 positive gated cells for the indicated cell surface markers. **a** Expression levels of CD68 (Gate FSC vs. SSC). **b** Expression levels of CCR7 (Gate CD68 positive cells). **c** Expression levels of CD163 (Gate CD68 positive cells). **d** Expression levels of CD206 (Gate CD68 positive cells). *WG* well ground, *CF* chitosan film (n = 4 for each condition, medians are indicated). *p < 0.05 and **p < 0.01 compared to WG at the same time point, ^###^p < 0.001 compared to all other samples at the same time point

In parallel to the changes in cell morphology, we detected changes in the expression of the following cell surface markers. (1) The macrophage differentiation marker CD68. (2) The chemokine receptor CCR7 antigen, present on the pro-inflammatory M1 macrophage subtype. (3) The scavenger receptor CD163 for which an increased expression is related to the M2a macrophage subtype, and (4) the CD206 alpha-mannose receptor, which is present on anti-inflammatory M2a and M2c macrophage subtypes [19]. The expression profiles of the analyzed markers are depicted in Figs. 9 and 10.

Results reveal that all macrophages independent of the different cultivation conditions expressed relatively constant levels of the macrophage differentiation marker CD68 (Figs. 9a, 10a).

The expression of the M1 macrophage marker CCR7 was not modulated before day 8 in culture (Figs. 9b, 10b), when a significant up-regulation was observed on non-stimulated cells cultured on WG. The lowest expression level was detectable in the M-CSF stimulated cultures on WG, while non-stimulated and stimulated cultures on CF showed a balanced expression level.

Analysis of the cell surface expression of CD163 revealed an upregulation on non-stimulated (significant from day 4 onward) and on M-CSF-stimulated macrophages seeded on WG (Figs. 9c, 10c). Macrophages cultured under both conditions on CF appeared to inhibit the up-regulation of this marker.

After 48 h in culture under non-stimulated conditions on CF, a strong upregulation of CD206 expression was detected (Figs. 9d, 10d). As expected, M-CSF induced an even more pronounced upregulation of this marker on macrophages cultured on regular WG (significant after 2 and 4 days in culture, Figs. 9d, 10d). Interestingly, the addition of M-CSF to macrophages cultured on CF did not strengthen the stimulatory effect, but in contrast a supposed explosive up-regulation was balanced to the same level seen in the non-stimulated CF condition (Figs. 9d, 10d). At later time points, i.e. 96 h and 8 days after plating, the differences between WG and CF related effects on the expression of CD206 under non-stimulated conditions were not detectable anymore (Fig. 10d). This is possibly an indirect effect due to the secretion of different cytokines by the cells and a subsequent attenuation of the biomaterial effect or the development of a homeostasis. Noteworthy is, however, that while M-CSF was still stimulating the CD206 expression on cells cultured on WG, chitosan kept on exerting the immunoregulatory effect when cells cultured on CF were supplemented with M-CSF (Fig. 10d).

## Discussion

Promising designs of novel nerve guides are usually examined in animal models using immediate reconstruction of peripheral nerve defects [20], but to our knowledge, none of the innovative designs has been recently tested after delayed nerve reconstructions in healthy and diabetic conditions. The diabetic condition represents an increasing prevalence in the human population and is inflicted with a reduced health and maintenance of the peripheral nervous system. In delayed nerve reconstructions, resection of scarred nerve tissue often results in an enlarged defect distance between the separated nerve ends. These kinds of defects have to be bridged by e.g. autologous nerve grafts. However, harvesting an uninjured nerve to support regeneration of a more important injured nerve trunk may not always be acceptable for the patient (e.g. prolonged surgery time, second surgery site, loss of function of the donor nerve). Therefore, nerve guides may be a suitable alternative to bridge shorter defects in delayed reconstruction; not only for the risk of residual problems from the donor side, but also for shortening the time for surgery. With regard to the systemic health conditions of the respective patients, it has to be considered that patients with diabetes may be in need of specific nerve guides others than those used for generally healthy patients. This is due to the fact that in diabetic neuropathy there are signs of both degeneration and regeneration (seen as regenerative clusters) and the general opinion is that nerve regeneration is impaired after a complete nerve injury and reconstruction in experimental systems [21]. Due to the potential increasing number of patients with diabetes, who have a broad spectrum of professional activities and may also injure their nerves by a trauma, any type of medical device should investigate the possibility to apply the device in diabetic conditions for which the diabetic GK rat represents an appropriate model resembling type 2 diabetes.

We have earlier demonstrated that the supportive properties of hCNGs are increased in CFeCNGs under immediate reconstruction conditions in both healthy and diabetic rats [10]. In the current study, we extended the focus to a comprehensive evaluation of the degree of early regeneration associated events and subsequent functional recovery after a delayed reconstruction of a 15 mm rat sciatic nerve gap with either one-chambered hollow hCNGs or two-chambered CFeCNGs in healthy Wistar rats and diabetic GK rats. The “deadline”, when a delayed nerve reconstruction in healthy or in diabetic conditions, irrespective of in experimental models or in clinical practice, can be done, is not clear. In the literature, it is not well defined what the best timing is to perform a study on delayed peripheral nerve reconstruction. We used a 45 days delay since it is sufficient time to induce biological effects in the peripheral (and central) nervous system with an impaired nerve regeneration process [22–24], but at a time point where there are minor risks for residual problems in the rats, e.g. risk for foot ulcers due to an unrepaired nerve injury. Furthermore, a term of 4 weeks up to 2 months of chronic denervation is, however, determined to be affiliated with considerable changes in the capacity of Schwann cells to support the regeneration process [25, 26]. The presently chosen delay period was based on these findings, and because it clearly exceeds the minimum period of 4 weeks (30 days). However, the generally insufficient nerve regeneration after a delayed nerve reconstruction can be attributed not only to an inability of Schwann cells to support axonal outgrowth, but also to alterations in the response of motor and sensory neurons to the injury [10, 24, 27].

In the current study, both examined nerve guides demonstrate their support of nerve regeneration across a critical defect length in healthy as well as in diabetic conditions even after a delayed nerve reconstruction, in spite of a variation in the formation of the regenerative matrix in the models as observed 56 days after reconstruction. As expected, the preoperative blood glucose levels positively correlated with the percentage of cleaved caspase-3 immunopositive Schwann cells in the distal nerve segment, indicating a higher risk for Schwann cells being apoptotic in conditions with higher blood glucose levels. The latter conditions appeared to be recapitulated in the delayed reconstruction scenario although the initial transection injury occurred 45 days before the reconstruction. During reconstruction, however, both nerve ends are manipulated (cut) again as 2 mm of the previously stitched up nerve ends are removed in order to insert clean nerve ends into the nerve guides. The HSP-27 expression in analyzed sections of dorsal root ganglia on the control side negatively correlated with the preoperative blood glucose levels as well as with the presence of cleaved caspase-3 immunopositive Schwann cells in the distal nerve segment, indicating a detrimental effect of a high blood glucose level and apoptotic cells on a neuroprotective substance. However, the diabetic condition did finally not affect the presence of axons in the distal nerve segment, as neurofilaments were present essentially to the same extent as in the healthy Wistar rats at 56 days after the delayed nerve reconstruction. This indicates that the condition of delayed nerve reconstruction did already modify the regenerative milieu and probably alleviate the difference between the healthy and the diabetic condition. We were only able to just detect (i.e. present or not) neurofilaments in the distal nerve segment. We could not securely measure the length of outgrowth or the amount of fibers to get a more detailed view of the regeneration in the short-term comparison [11, 14] because longitudinal sections were prepared in order to primarily analyze ATF-3 and cleaved caspase-3 immunopositive cells in the distal nerve segment. Furthermore, we could not follow all the neurofilaments across the conduits due to the variations in the formed regenerative matrices (i.e. single- and double-strand matrices or non-complete matrix; in the latter probably axonal growth along the wall of the conduit). Irrespective of this, we observed neurofilament immunopositive axons in the distal nerve segment indicating that the use of chitosan nerve guides is as valid in diabetic as in healthy conditions.

Similar to our previous findings [10], through the long-term observation we detected again an advantage of CFeCNGs over hCNGs with regard to the support of overall tissue regeneration and percentage of animals per group displaying functional motor recovery. The onset and progress of functional recovery and its degree, however, are considerably retarded and less complete, respectively, than after immediate nerve reconstruction. This indicates again the importance of prompt nerve reconstruction after injury [22, 28]. The course of functional recovery detected in the current study is likely related to the thin regenerative matrix formed during the 56 days after delayed nerve reconstruction of the 15 mm critical defect lengths with either type of nerve guide examined, i.e. hCNG or CFeCNG. The formation of a regenerative matrix crucially determines the outcome of peripheral nerve regeneration through bioartificial nerve guides [15, 29]. Our results indicate that this formation of a regenerative matrix is disturbed after a delayed nerve reconstruction both in healthy and diabetic conditions.

We observed a higher number of activated ATF-3 as well as apoptotic cleaved caspase-3 immunopositive Schwann cells after delayed nerve reconstruction (current study) compared to immediate nerve reconstruction across this extended nerve defect (15 mm) even if the evaluation period was similar, i.e. 56 days post-surgery [10]. We have no explanation for this observation other than that the manipulation of the nerve ends during surgical nerve reconstruction could have influenced the cell behavior. In general, one would expect a lower percentage of stained Schwann cells, as verified by the double-staining (Additional file 2: Figure S2) and the previously used method of analysis [22, 23], after a delayed nerve reconstruction [27]. A low percentage of activated Schwann cells is due to a transient upregulation of different factors supporting nerve regeneration over time with subsequent impaired ability of Schwann cells to stimulate regeneration after a delayed nerve reconstruction [22, 24, 30]. The proportions of ATF-3, i.e. activated, and cleaved caspase-3, i.e. apoptotic, stained Schwann cells probably balance each other in numbers; thus, if more activated cells are available in a nerve segment the more apoptotic cells may occur [11, 14], although no positive correlation could be detected in the current study.

Furthermore, even if neurofilament immunopositive axons were present in the distal nerve segment of all rats in the groups examined 56 days after the delayed reconstruction, the regrowing axons appeared to be less sufficient or too low in numbers to properly regain function in comparison to the conditions after an immediate nerve reconstruction. Indeed, the number of myelinated axons detected in nerve morphometry of distal nerve segments after long-term evaluation in the current study was reduced to approximately 50% of the values detectable after immediate nerve reconstruction [10]. With regard to functional recovery, the tibialis anterior muscle CMAP amplitude ratio, for example, recovered to about >20–40% in more than 50% of the sciatic nerves immediately reconstructed with CFeCNGs [10]. After a 45-day delay of reconstruction, we found a recovery of only 10–30% for most sciatic nerves reconstructed with CFeCNGs. This, again, indicates the importance of a timely reconstruction of injured peripheral nerves [22, 24, 30]. Whenever immediate nerve reconstruction is not an option, however, the nerve guides used for a delayed approach should still support the regeneration process.

Indeed, our immunohistological results indicate a specific promotion of the regrowth of ChAT-immunopositive motor axons by the chitosan film centrally introduced to the CFeCNGs. This finding could eventually be strengthened by the morphometric results displaying a slightly increased diameter of axons regenerated through CFeCNGs. Although it has already been demonstrated a few years ago that introducing a central biomaterial-film into a hollow tube can improve nerve regeneration through the same [31], it is very likely that the specific chitosan film used within the CFeCNGs provided a specific growth support. Specific differentiation of the macrophage population into a M2-phenotype dominated population of regulatory and healing macrophages at the injury site or within the nerve guide [32] has been accounted responsible for improved peripheral nerve regeneration. Furthermore, 3D scaffolds made out of chitosan with a similar degree of ~5% acetylation as of the chitosan material applied in the current study, provoked an inflammatory response after their subcutaneous implantation in mice that was characterized by high numbers of reparative M2 macrophages adherent to the scaffolds and increased levels of anti-inflammatory cytokines within the exudates at the implantation site [13]. Several studies indicate that biomaterials can provide an immunomodulatory stimulus [32] and that their biophysical and mechanical properties impact the differentiation and subsequent polarization of macrophage subtypes [33] within the pro- or anti-inflammatory response to tissue injury. Macrophage subtypes represent distinct functional phenotypes that are continuously formed in response to changes in the cells microenvironment [33]. When arriving from the peripheral blood at the site of a peripheral nerve injury or in the distal nerve end, an inherent pro-regenerative milieu develops that induces an M2 phenotype [18, 34]. This represents a first step towards axon and Schwann cell modulations that can finally lead to stimulation of the peripheral nerve regeneration process not only in the distal nerve end, but also in a nerve conduit or in an autologous nerve graft [32, 35].

Our in vitro results provide an insight into the mechanism(s) by which the chitosan biomaterial can specifically support the peripheral nerve regeneration process when introduced into chitosan nerve guides as a central chitosan film. Although we are not the first describing the immunomodulatory effect of chitosan, others have been investigating it in the context of subcutaneous implantable device surfaces [13, 19]. In accordance to these earlier data, we clearly demonstrate that our chitosan films trigger the differentiation of monocyte-derived macrophages from human blood samples towards the pro-regenerative M2 phenotype. Macrophages [35], and in particular the M2 macrophage phenotype, have previously been attributed to provide crucial support for peripheral nerve regeneration [12]. Furthermore, chitosan with a degree of acetylation of 5%, similar to that in our study, was specific in this pro-regenerative immunomodulation after subcutaneous application of the biomaterial, while a higher degree of acetylation induced a pro-inflammatory response [13]. Also, in line with previous results [19] from in vitro analysis of human primary monocyte-derived macrophages cultured on chitosan films with a degree of acetylation of ~10%, we demonstrate high expression of CD206 paralleled by a low expression rate of CD163 in our cells, indicating that chitosan specifically triggers the M2c subtype polarization. While CD206 is expressed on both the M2a and M2c subtype, CD163 is expressed more on M2a macrophages [19]. Both subtypes are likely to support peripheral nerve regeneration through our chitosan nerve guides because both are known to secrete growth factors to support angiogenesis and tissue regeneration [33]. Here and earlier [10], we demonstrated that the regenerated tissue formed in CFeCNGs is better vascularized in comparison to that developed in hCNGs. Furthermore, the M2c macrophage subtype has an impact on the extracellular matrix remodeling [33], and this event is crucial during the early phase of peripheral nerve reconstruction with nerve guides. As already mentioned above, the successful formation of the regenerative matrix is needed for successful peripheral nerve regeneration [15, 29] and again, the current study demonstrates that this process is supported by CFeCNGs in comparison to hCNGs not only after immediate nerve reconstruction [10] but also after a delayed nerve reconstruction.

## Conclusions

The presented results clearly indicate that, in comparison to hCNGs, CFeCNGs provide an increased support of motor axon regeneration after delayed nerve reconstruction. The tested nerve guides alone are, however, not sufficient to support a degree of axonal and functional motor recovery similar to that after immediate nerve reconstruction. It is, however, a known fact that nerve regeneration after delayed reconstruction is impaired [36, 37] and the possible strategies to improve it range widely from targeting molecular cues [38] to supplementary treatment strategies, like intra-surgical electrostimulation or rehabilitation exercise [36].

To more deeply analyze the mechanism(s) behind the specific support of motor axon regeneration by the chitosan material in CFeCNGs, and not in hCNGs, could, however, provide helpful indications for future modifications of the nerve guides in order to make them qualified products also for delayed nerve reconstruction in healthy and diabetic conditions with the intention also to improve the formation of the regenerative matrix.

Obviously, not only the biochemical, but also the biophysical and mechanical cues, provided by the biomaterial used for peripheral nerve reconstruction have to be carefully concerned in healthy and diabetic conditions when tissue engineering approaches should lead to an optimized remodeling of the physiological conditions provided by autologous nerve grafts. Chitosan, however, represents a valuable biomaterial for future research as it has clearly demonstrated also significant immunomodulatory effects.

With regard to a potential need of nerve guides specific for patients with diabetes, our current results indicate that this cannot be focused on further at this stage of research, because the delayed nerve reconstruction condition did not result in considerable differences between healthy and diabetic subjects.

## Methods

### Manufacturing of one-chambered hollow chitosan nerve guides (hCNGs) and two-chambered chitosan film enhanced chitosan nerve guides (CFeCNGs)

Chitosan was extracted from Pandalus borealis shrimp shells and further processed to certified medical grade by Altakitin S.A. (Lisbon, Portugal). Afterwards, hollow chitosan nerve guides (hCNGs; length: 19 mm, inner diameter: 2.1 mm; REAXON^®^ Nerve Guide) and chitosan films (CFs) were produced according to ISO 13485 requirements by Medovent GmbH (Mainz, Germany) with a final degree of acetylation (DA) of ~5% as previously described [7]. Chitosan film enhanced chitosan nerve guides (CFeCNGs) were manually made up as described earlier [10]. In brief, they are composed of rectangular pieces (length: 15 mm, width: 5 mm) of perforated (along the midline of the longer axis, diameter of perforation: 0.3 mm, distance in between: 2 mm) and subsequently Z-shape-folded CFs (opposite kinked edges, width of outer edges: 1.5 mm) that were introduced into the lumen of hCNGs leading to 2 mm spaces on each site. Before application, hCNGs and CFeCNGs were sterilized by electron beam.

### Animals and surgical procedure

All experimental protocols were approved by the local authorities (Animal Ethics Committee in Malmö/Lund, Sweden, reference number M-347-11 and M131-14, and animal care committee of Lower-Saxony, Germany: Nds. Landesamt für Verbraucherschutz und Lebensmittelsicherheit Dezernat 33/Tierschutz, reference number 33.12 42502-04-12/0816).

The animal experiments were performed in two different laboratories [Dahlin lab at Lund University (ULUND), Sweden, and Grothe lab at Hannover Medical School (MHH), Germany] using different animal breeders and regimes for anesthesia and analgesia based on different national rules for the care and use of laboratory animals. In common, adult female Wistar rats with an average weight of 220–285 g were housed in pairs of two animals (MHH) or in groups of four (ULUND) under standardized housing conditions [room temperature 22.2 °C; humidity 55.5%; light–dark cycle 14 h/10 h (MHH) or 12 h/12 h (ULUND)]. Adult female Goto-Kakizaki rats (GK rats) were kindly provided by Malin Fex, Lund University, Sweden, with an average weight around 230 g. Food and water was provided ad libitum, with providing extra water for the diabetic GK rats at ULUND. At MHH, water was supplemented with amytriptiline hydrochloride (13.5 mg/kg/day, Amitriptylin-neuraxpharm^®^; Neuraxpharm Arzneimittel GmbH, Germany) during the whole experiment starting 4 weeks prior to the initial transection surgery to reduce the possibility of occurring automutilation as known from literature [39]. Once weekly, fasting blood sugar was determined in the rats at ULUND. Therefore blood harvested from the tail vein was analyzed [Ascensia contour™ (Bio health Care, USA, Bio Diagnostics Europe) and LT (Bayer AB, Diabetes Care, Solna, Sweden); test slips Microfil™ (Bio Healthcare Diabetes Care, USA)].

All surgical interventions and non-invasive electrodiagnostic recordings were performed under aseptic conditions with sufficient anesthesia and analgesia applied. At ULUND, anesthesia was induced by intraperitoneal injection of Rompun (20 mg/ml, Bayer Health Care, Germany) and Ketalar (10 mg/ml, Pfizer, Finland) at a dose of 0.125 ml/kg bodyweight. Post-operative pain was relieved by intramuscular injection of buprenorphine of 0.01–0.05 mg/kg bodyweight (0.3 mg/ml, Temgesic^®^; Schering-Plough Europe, Belgium). At MHH, anesthesia was induced by intraperitoneal injection of chloral hydrate (370 mg/kg, Sigma-Aldrich Chemie GmbH, Germany) and for surgical interventions, sufficient analgesia was ensured by locally applied bupivacaine (0.25%, Carbostesin^®^; AstraZeneca GmbH, Germany) and lidocaine (2%, Xylocain^®^; AstraZeneca GmbH, Germany) on the exposed nerve and subcutaneous injection of butorphanol (0.5 mg/kg, Torbugesic^®^; Pfizer GmbH, Germany) at the day of surgery and two post-operative days. For electrodiagnostic measurements, sufficient analgesia was achieved by intramuscular injection of buprenorphine (0.045 mg/kg, Temgesic^®^; Bayer Vital GmbH, Germany).

For nerve transection surgery, the left sciatic nerve was exposed at mid-thigh level as previously described [7, 10, 11] and cut with micro-scissors at a constant level (2.5 mm distal to the aponeurosis) without removing any nerve tissue. To prevent spontaneous recovery, both nerve ends were sutured in a loop-like shape (Additional file 1: Figure S1, 9-0 Ethilon, EH7981G, Ethicon, Scotland) resulting in an initial gap of 6 mm in between the nerve ends. After 45 days, the transected sciatic nerve was re-exposed and the nerve ends found to be approximately 11 mm apart from each other. The nerve ends were each refreshed by resecting 2 mm of the scar tissue loop and the nerve ends sutured with one epineurial stitch at each end (9-0 Ethilon) into the respective nerve guide with an overlap of 2 mm. Before nerve reconstruction with either hCNG or CFeCNG, the nerve guides were softened in 0.9% sodium chloride solution (NaCl 0.9%, B. Braun Melsungen GmbH, Germany) for at least 20 min.

### Analysis of the regenerative matrix at 56 days after delayed nerve reconstruction (study I)

In study I, the reconstructed sciatic nerve was dissected together with the used nerve guide at 56 days after delayed nerve reconstruction. The contents of the nerve guides (e.g. the regenerative matrix, if formed) together with the respective proximal and distal nerve ends were processed for sectioning using a cryostat as described before [7, 10, 11, 14]. Blind-coded, longitudinal sections at 4 µm thickness were collected on Super Frost^®^ plus glass slides (Menzel-Gläser, Germany) and immunohistology was performed to evaluate (1) presence of axons by neurofilament staining [anti-human neurofilament protein (DAKO Glostrup, Denmark), 1:80 in 0.25% Triton-X 100 and 0.25% FCS in phosphate buffered salt solution (PBS)/Alexa Fluor 594 conjugated goat anti-mouse IgG (Invitrogen, Molecular Probes, USA), diluted in 1:500 in PBS], and (2) activated Schwann cells, and (3) apoptotic Schwann cells, respectively, by anti-activating transcription factor 3 (ATF-3) and anti-cleaved caspase-3 staining [rabbit anti-ATF-3 polyclonal antibody (1:200; Santa Cruz Biotechnology, USA) or anti-cleaved caspase-3 antibody (1:200; Invitro Sweden AB, Stockholm, Sweden); both diluted in 0.25% Triton-X 100 and 0.25% FCS in PBS/Alexa Fluor 488 conjugated goat anti-rabbit IgG (Invitrogen, Molecular Probes, USA), diluted in 1:500 in PBS]. The Schwann cells were identified on their location and the oval shaped nuclei [14, 30]. Furthermore, double-staining for ATF-3 or cleaved caspase-3 and S-100 was additionally performed in single experiments as earlier described [30]. Finally, the slides were mounted with 4′,6′-diamidino-2-phenylindole DAPI (Vectashield^®^, Vector Laboratories, Inc. Burlingame, USA) to visualize the nuclei (i.e. for counting the total number of the cells) and cover slipped.

As earlier described [7, 10, 11, 14], in digitalized sections, the presence of outgrowing axons was classified as present or non-present in three randomly selected sections in the distal nerve segment just distal to the distal suture line. Cells immunopositive for cleaved caspase-3 and ATF-3 were counted in one section (image size 500 × 400 µm) at three different levels in the matrix and in the adjacent sciatic nerve: at 3 mm from the proximal nerve suture, in the center of the formed matrix in the nerve guides and in the distal nerve segment. The same squares were also used for counting the total number of DAPI stained cells (no/mm^2^). The images (20× magnification) were analyzed with NIS elements (Nikon, Japan).

### Assessment of sensory dorsal root ganglia at 56 days after delayed nerve reconstruction (study I)

Together with harvest of the nerve guide content at 56 days after delayed nerve reconstruction, the dorsal root ganglia (DRGs) L4 and L5 were collected bilaterally and processed on the cryostat into longitudinal sections (8 µm thickness) collected on Super Frost^®^ plus glass slides (Menzel-Gläser, Germany). The DRG sections were air dried, washed in PBS for 15 min, and thereafter incubated for ATF-3 immunohistology (protocol as above) or incubated with a primary goat-anti-Heat Shock Protein 27 antibody [HSP-27, sc-1048, Santa Cruz Biotechnology, USA; dilution 1:200 in 0.25% Triton-X 100 (Sigma-Aldrich, USA) and 0.25% bovine serum albumin (BSA; Sigma-Aldrich, USA)] in PBS overnight at 4 °C. The anti-HSP-27 antibody was detected with the secondary Alexa Fluor 488 donkey anti-goat antibody (Molecular Probes, Eugene, Oregon, USA; dilution 1:500) in PBS for 2 h at room temperature followed by a further wash with PBS for 3 × 5 min. Finally, these sections were cover-slipped with Vectashield^®^ (Vector Laboratories, CA, USA) containing DAPI for counterstaining of the nuclei for evaluation of cell activation (i.e. ATF-3) and presence of the neuroprotective substance Heat Shock Protein 27 (HSP-27).

Photomicrographs were taken at 20× magnification and imported into ImageJ (http://imagej.nih.gov/ij/) in order to analyze the presence of ATF-3 activated sensory neurons and the expression of the neuroprotective HSP-27 in the DRGs as earlier described [10, 22]. ATF-3 stained sensory neurons were quantified and expressed as percent of total number of sensory neurons (i.e. DAPI stained cells). A region of interest (ROI) covering 75 × 75 pixels was determined to analyze HSP-27 expression. The tool threshold was used to determine the immunolabelling with the intensity threshold decided by adding three times the standard deviation of the background to the mean intensity (${\bar{\text{x}}}$ + 3 × SD). Measurement of the immunopositive area was performed across the entire section and expressed in percent of the total area of the section containing cell bodies; thus both, intensity of neurons and their satellite cells, as well as in thin axons, were included. Furthermore, the HSP-27 expression was also expressed as a ratio; i.e. percent HSP-27 at the experimental side divided by the expression on the control side [10].

### Assessment of functional motor recovery over a period of 150 days after delayed nerve reconstruction (study II)

#### Non-invasive electrodiagnostic recordings

Non-invasive electrodiagnostic recordings were performed as described earlier [7, 10] every 30 days after the delayed reconstruction surgery starting on 60 days post-reconstruction. In short, the animals were anesthetized, placed in prone position and the body temperature kept on a constant level. Transcutaneous monopolar needle electrodes delivered single electrical impulses (100 µs duration with supramaximal intensity at 1 Hz frequency) to the reconstructed sciatic nerves either at the sciatic notch (proximal stimulation site) or into the popliteal fossa (distal stimulation site). Compound muscle action potentials (CMAP) were recorded for both sides of each individual (left reconstructed side and healthy side as control) from the tibialis anterior (TA) or the plantar interosseus muscles (PL) using a Keypoint portable system (Natus Europe, Germany). To evaluate the degree of reinnervation, latencies and amplitudes of the evoked CMAPs were taken into account and ratios calculated (lesioned side divided by non-lesioned side). If no evoked CMAPs could be detected, amplitude ratios were set to “0” value but still included into the statistical analysis.

#### Muscle weight ratio

After final functional analysis at 150 days after delayed nerve reconstruction, the animals were sacrificed and the tibialis anterior and gastrocnemius muscles harvested from both sides and weighted. The muscle weight ratio of each animal was calculated by muscle weight [g] on lesioned side divided by muscle weight [g] on healthy side. All values were included in statistical analysis irrespectively of the results obtained in either electrodiagnostic measurements or individual macroscopic regeneration outcome.

### Nerve immunohistology and histomorphometry 150 days after delayed nerve reconstruction (study II)

#### Nerve (immuno-)histochemistry

After final assessment of functional motor and sensory recovery, animals were sacrificed and the nerve guides together with the regenerated tissue were harvested for further investigation. The macroscopic appearance of the regenerated nerve tissue was assessed after removing the chitosan nerve guides. Afterwards, the nerve tissue (in case of CFeCNGs together with the central chitosan films) was fixed overnight in 4% paraformaldehyde in PBS at 4 °C for subsequent paraffin-embedding and (immuno-)histological evaluations. One series of eighty blind-coded sections with a thickness of 7 µm each was prepared within the distal part of the regenerated tissue (560 µm segment from 12.7 to 13.3 mm distal to the original proximal nerve end). The sectioning was initiated 3.7 mm proximal to the distal end of the nerve guides and continued in proximal direction. Therefore, in case of CFeCNGs, the sections included the most distal perforation within the chitosan films. To visualize the general tissue histology, sections were subjected to trichrome (collagen) staining as described before [10]. In brief, sections were consecutively processed with hemalum solution acid according to Mayer (Roth, Germany), staining solution containing acetic acid (1% in H_2_O) and acetic fuchsine (0.5% in 1% acetic acid, both Merck, Germany) mixed with light green (1% in 1% acetic acid, Chroma Gesellschaft, Schmidt & Co., Germany) and wolframite phosphoric acid (1% in H_2_O, Merck, Germany), and finally 1% acetic acid.

For immunohistology, sections successive to the ones processed in trichrome staining were double-stained for neurofilament and choline acetyltransferase (ChAT) as previously described [10]. Briefly, as first blocking solution we used in 5% horse serum diluted in PBS prior to incubation with the following primary-secondary anti-body combination: goat anti-ChAT antibody (1:50, in blocking solution, AB144P, Millipore, Germany) detected by Alexa Fluor 555-conjugated donkey anti-goat antibody (1:500, in blocking solution, A21422, Invitrogen, Germany). The second blocking solution was 3% milk powder/0.5% Triton-X 100 in PBS. Primary rabbit anti-NF200 antibody (against phosphorylated NFeH, 1:500, in blocking solution, N4142, Sigma-Aldrich, Germany) was detected by Alexa Fluor 488-conjugated goat anti-rabbit antibody (1:1000, in blocking solution, A11034, Invitrogen, Germany). Nuclear counterstaining was achieved with DAPI (1:2000, in PBS, Sigma-Aldrich, Germany).

Representative photomicrographs of the processed and mounted sections were taken as multiple image alignments (MIA) with the help of a BX51 microscope (Olympus, Germany) expanded with a joystick controlled microscope stage (MBF Bioscience, USA) and the Stereo Investigator software version 11.07 (MBF Bioscience, USA). Exposure time was adjusted for each staining and channel and held constant for microscopy of different sample sections making them comparable in a quantitative manner. For the quantification of NF200- and ChAT-immunopositive profiles, ImageJ version 1.48 (National Institutes of Health, USA) was applied. NF200-immunopositive areas were employed to define the regions of interest (ROI), which were later used for quantification of particle number. Next, binary images were generated from NF200-photomicrographs using a threshold strategy allowing for distinction of individual nerve fibers. The same threshold was applied to all samples. Similarly, ChAT-images were transformed to binary images. Now, the particle analysis function of ImageJ was used for detection of NF200-positive nerve fibers within the ROI. Noise was reduced by excluding particles with a size smaller than 0.001 Pixel^2^. Similarly, motor neuron fibers, which are positive for both, NF200 and ChAT, were detected on a binary image generated by multiplication of the NF200- and ChAT-binary images using the image calculator function of ImageJ. This allows for exclusion of unspecific ChAT-immunopositive signals from the quantification process. Only animals showing an evocable CMAP were included and analyzed at the same site of the regenerated tissue.

### Resin embedding and electron microscopic analysis

Distal nerve segments (5 mm long tissue samples harvested from the nerve directly distal to the nerve guides) were fixed by immediate immersion in glutaraldehyde (2.5% in 0.1 M phosphate buffer saline pH 7.4) for 4–5 h at 4 °C. Samples were then post-fixed in 2% osmium tetroxide for 2 h and dehydrated through a graded series of ethanol from 30 to 100%. Thereafter, samples were infiltrated with propylene oxide (two passages of 7 min each) and then immersed in a 1:1 mixture of propylene oxide and Glauerts’ mixture of resins (made of the same part of Araldite M and the Araldite Harter, HY 964) overnight. The following day, samples were embedded in Glauerts’ mixture of resins where 0.5% of the plasticizer dibutyl phthalate and 2% of accelerator 964 were added. Nerve samples were finally oriented in embedding molds and left 48 h at 60 °C for polymerization.

Semi-thin sections of 2.5 µm were cut using an Ultracut UCT ultramicrotome (Leica Microsystems, Wetzlar, Germany) and stained with 1% toluidine blue. A DM4000B microscope equipped with a DFC320 digital camera and an IM50 image manager system (Leica Microsystems, Wetzlar, Germany) was used for section analysis.

Ultrathin sections of 70 nm were cut with a diamond knife (Diatome, UK) and collected on copper grids coated with Pioloform film. Sections were counter-stained with saturated aqueous solution of uranyl acetate and lead citrate. Sections were finally analyzed using a JEM-1010 transmission electron microscope (JEOL, Tokyo, Japan) equipped with a Mega-View-III digital camera and a Soft-Imaging-System (SIS, Münster, Germany) for the computerized acquisition of the images.

### Stereology and quantitative morphometry

Parameters related to nerve regeneration (mean fiber density, total fiber number, fiber and axon diameter, myelin thickness and g-ratio) were estimated at electron microscopic level using a protocol previously described [40]. We randomly selected three nerve specimens of each group from animals with motor recovery detectable as evoked CMAP recorded from the tibialis anterior muscle. Briefly, one randomly selected ultra-thin section was chosen and 20–25 fields were selected using a systematic random sampling protocol (magnification of 4000×). Then, in each sampling field, a two-dimensional dissector procedure was adopted to count (and then measure) myelinated fibers [41]. The total cross-sectional area of the whole regenerated nerve was obtained on a semi-thin section (with 10× of magnification).

### Evaluation of macrophage polarization on chitosan films

To get an insight into the mechanism by which chitosan films inserted into the CFeCNGs could support peripheral nerve regeneration, additional in vitro experiments have been conducted. These experiments focused on the role of macrophages within the regeneration process.

### Material preparation

Sterile chitosan films (CF) with a DA of 5% were cut in squares of 2 × 2 cm and fixed in cell crowns (CellCrown™24, Scaffdex^®^, Finland) for later insertion into 24-well plates (Nunclon™ Delta Surface, Thermo Fischer Scientific, Denmark). First, the fixed CFs were washed 3 times for 30 min at room temperature in sodium chloride (NaCl 0.9%, B. Braun Melsungen GmbH, Germany). Afterwards, the washing solution was replaced by macrophage generation medium composed of RPMI 1640, supplemented with 2 mM l-glutamine, 100 mg/ml penicillin/streptomycin, 12 mM Hepes, and 5% v/v FCS (PAN-Biotech; all other media components from Biochrom, Berlin, Germany). CFs were soaked in the macrophage generation medium for 72 h in the incubator (37 °C, 5% CO_2_; Sanyo CO_2_ Incubator, Ewald Innovationstechnik, Germany) before initial seeding of the cells.

### In vitro differentiation of human monocyte derived macrophages

Residual blood samples from platelet (PLT) apheresis disposables used for routine PLT collection and of regular anonymous healthy donors served as source material for the isolation of human peripheral blood mononuclear cells (PBMCs). PBMCs were separated by density gradient centrifugation on lymphoprep (Pancoll, PAN-Biotech, Aidenbach, Germany). To examine the influence of chitosan on the differentiation process of human macrophages, the PBMCs were plated with a seeding density of 1 × 10^6^ cells pro well in a 24-well cell culture polystyrene plate (non-coated bottom of 24-well cell culture plate, regular well ground, WG, Nunclon™ Delta Surface) on plain well grounds or on the inserted soaked CFs in an appropriate amount of macrophage generation medium. To attach the monocytes, the cells were incubated for 2 h at 5% CO_2_ and 37 °C. The non-adherent cells were removed by vigorously washing the adherent cells three times with phosphate buffered saline (PBS, Merck KGaA, Germany) and 1 ml macrophage generation medium was added. The cells were incubated for 4 days at 5% CO_2_ and 37 °C without medium change. In one approach, the macrophages either attached on the well ground or on CFs were differentiated in macrophage generation medium supplemented with macrophage-colony stimulating factor (M-CSF) (10 ng/ml) (R&D, Wiesbaden, Germany), a cytokine which promotes the M2 phenotype. At day 4, another 70% by volume of fresh medium was added. At day 7, the medium was completely changed.

### Microscopic evaluation of cell morphology

Every 24 h, cell growth was examined using a phase contrast microscope (Olympus IX 70 microscope, Olympus, Germany) and changes in morphology were recorded using CellP software (Olympus, Germany).

### Flow cytometric analysis of macrophage differentiation and cell surface markers

At day 2, 4 and 8 the differentiated macrophages were carefully scraped from the CFs or well ground of the culture plates and seeded (5 × 10^5^ cells per well) into 96-well plates. Cells were washed with PBS. Subsequently the Fc receptors were blocked by incubation in a buffer containing 10 µg/ml heat-aggravated human immunoglobulin G (IgG) (Sigma, Deisenhofen, Germany). For extracellular staining the following antibodies were used: anti-CD163-PE (BioLegend, San Diego, CA, USA), anti-CD206-PE, (BD Pharmingen, Heidelberg, Germany) and anti-CCR7-FITC (R&D Systems) and the respective isotype controls to define the unspecific background staining. For intracellular staining cells were fixed, permeabilized using the BD Cytofix/Cytoperm fixation/permeabilization kit (BD Bioscience, Heidelberg, Germany) and anti-CD68-APC (BioLegend) or the respective isotype control was applied. Stained cells were washed three times and resuspended in PBS. Sample acquisition was performed by flow cytometry (FACS Calibur, Becton Dickinson, Heidelberg, Germany) and geometric mean fluorescence intensities (MFI) were calculated by CellQuest Pro software (Becton Dickinson).

### Statistics

Data obtained in the different studies were subjected to statistical analysis. For the short-term experiments (software IBM SPSS Statistics, version 22) results are presented as median values [with 25th–75th percentiles] due to the non-normal distribution of data. The Kruskal-Wallis test was used to determine any significant difference between the four groups, while the Mann–Whitney post hoc test was used to specifically identify differences between the four groups only if the Kruskal–Wallis p-value was <0.05: hCNG-I^healthy^, CFeCNG-I^healthy^, hCNG-I^diabetic^ and CFeCNG-I^diabetic^. Separate p-values from the Mann–Whitney post hoc test for healthy/diabetic rats and hCNG-I/CFeCNG-I were then combined to a single p-value by using the Fisher method for independent samples based on the Chi square distribution [42, 43], courtesy by statistician Professor Jonas Björk, Lund University, Lund, Sweden [14]. Spearman rank correlation test was used to evaluate the association between specific different parameters (expressed as rho-values and p-values). A p-value of less than 0.05 was considered significant.

For the long-term experiments two-way ANOVA (Analysis of Variance) followed by Tukey’s multiple comparison was used [GraphPad Prism for Windows in version 6.05 (GraphPad Software, USA)]. The proportion of animals per group that displayed a predefined qualitative parameter (evocable CMAP or macroscopically visible tissue regeneration) was calculated as percentage (0–100%) and analyzed with the Chi square test to elucidate differences between the hCNG-II and CFeCNG-II group. As level of significance, the p-value was set at <0.05.

Statistical analysis of the results obtained in vitro was performed using two-way ANOVA (Analysis of Variance) followed by Tukey’s multiple comparison [GraphPad Prism for Windows in version 6.05 (GraphPad Software, USA)].

## Additional files

**Additional file 1: Figure S1.** Suture technique to prevent spontaneous recovery of free nerve ends. (A) Photograph of one nerve end sutured in the described loop-like shape. The scale paper in top of the picture indicates the dimensions of the sutured nerve end in mm. (B) Stepwise illustration of the suture technique. The first puncture was made through the epineurium around 2 mm next to the nerve end exiting at the medial transection level. The second puncture was entering only the epineurium of the nerve and exiting parallel to the transection site. Finally the suture was tied up to generate the loop-like shape of the nerve end.

**Additional file 2: Figure S2.** Detection of ATF-3 or cleaved caspase-3 stained Schwann cells in distal nerve segments after reconstruction. Photomicrographs present distal nerve segments at 56 days after 45 days delayed reconstruction double-stained for ATF-3 (A-D, in green) or cleaved caspase-3 (E-H, in green) with S-100 (A-H, in red). The inserts in A and E show a detail of a double-stained Schwann cell (S-100-immunopositive, red) with enclosed ATF-3 (green) or cleaved caspase-3 (green), respectively. Abbreviations: hCNG-I^healthy^ = hollow chitosan nerve guide from healthy rats; CFeCNG-I^healthy^ = chitosan film enhanced chitosan nerve guide from healthy rats; hCNG-I^diabetic^ = hollow chitosan nerve guide from diabetic GK rats; CFeCNG-I^diabetic^ = chitosan film enhanced chitosan nerve guide from diabetic rats. For detailed results of the quantification see Table 2. Scale bars display 100 µm in all images.

**Additional file 3: Figure S3.** Quantitative analysis of motor recovery over 150 days post delayed reconstruction. As depicted by dot plots, the recovery of the evoked compound action muscle potential (CMAP) amplitude ratios (A) and the nerve conduction velocity ratios (B) displayed no significant quantitative difference between the two groups over the 150 day observation time after delayed nerve reconstruction. The lower limb muscle weight ratio (C) as determined at the endpoint of the study (150 days) did also not reveal any significant quantitative difference between the groups. Error bars indicate median ± range.

**Additional file 4: Figure S4.** Representative high resolution images of ultrathin cross-sections. Photomicrographs show distal nerve segments of animals without an evocable CMAP, hCNG-II (A) and CFeCNG-II (B), in contrast to samples of animals with evocable CMAPs, hCNG-II (C) and CFeCNG-II (D), 150 days after reconstruction. Scale bar displays 5 µm for all pictures.

## Abbreviations

ATF-3: activating transcription factor 3
BSA: bovine serum albumin
CCR7: chemokine receptor antigen
CD68: macrophage differentiation antigen
CD163: scavenger receptor
CD206: alpha-mannose receptor
CF: chitosan film
CFeCNG: chitosan film enhanced chitosan nerve guide
ChAT: choline acetyltransferase
CMAP: compound muscle action potential
DAPI: 4′,6′-diamidino-2-phenylindole
DRG: dorsal root ganglion
FACS: flow cytometry
FCS: fetal calf serum
hCNG: hollow chitosan nerve guide
HSP-27: Heat Shock Protein 27
GK: Goto-Kakizaki
M-CSF: macrophage-colony stimulating factor
MFI: geometric mean fluorescence intensities
MHH: Medizinische Hochschule Hannover, i.e. Hannover Medical School
MIA: multiple image alignments
NA: not applicable
NF200: neurofilament 200
KW: Kruskal–Wallis
PBMC: human peripheral blood mononuclear cell
PBS: phosphate buffered saline
PL: plantaris interosseous muscle
PLT: residual blood samples from platelet
SND: distal nerve segment
TA: tibialis anterior muscle
TFO: too few observations to apply statistics
ULUND: University of Lund
UNITO: University of Turin
WG: well ground, i.e. regular cell culture surfaces
