# Supplementary figures and images for: Regeneration of long-distance peripheral nerve defects after delayed reconstruction in healthy and diabetic rats is supported by immunomodulatory chitosan nerve guides
Source: BMC Neurosci. 2017 Jul 18;18:53. doi: 10.1186/s12868-017-0374-z (PMC5516317; doi:10.1186/s12868-017-0374-z)

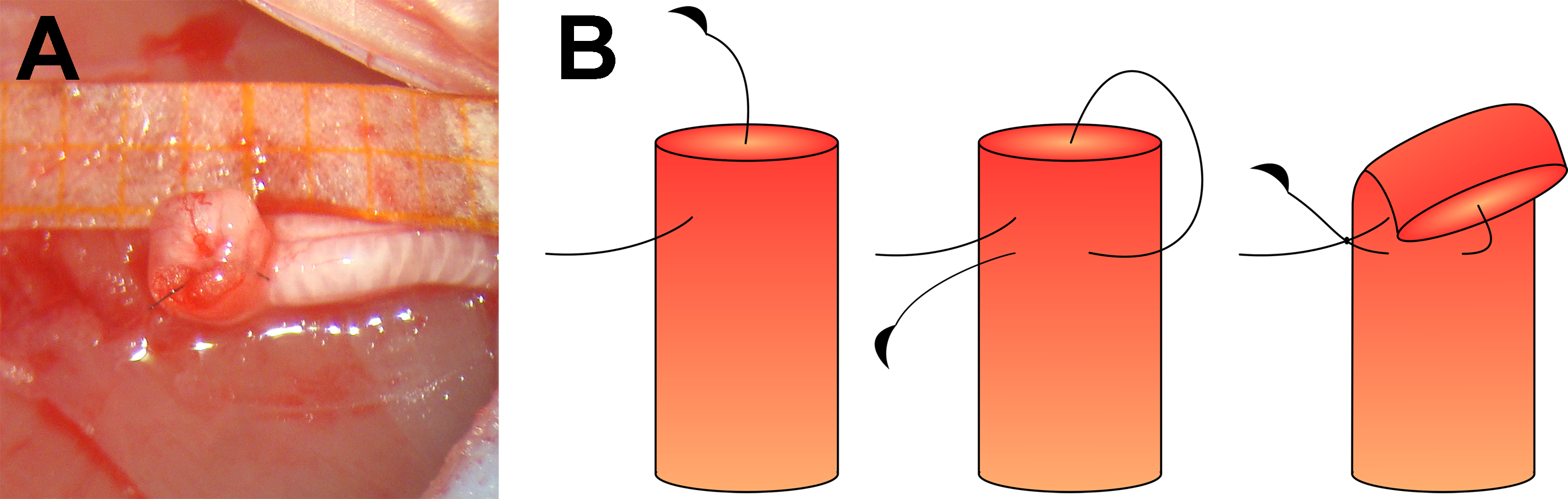

Supplement: Supplementary file 1 — Additional file 1: Figure S1. Suture technique to prevent spontaneous recovery of free nerve ends. (A) Photograph of one nerve end sutured in the described loop-like shape. The scale paper in top of the picture indicates the dimensions of the sutured nerve end in mm. (B) Stepwise illustration of the suture technique. The first puncture was made through the epineurium around 2 mm next to the nerve end exiting at the medial transection level. The second puncture was entering only the epineurium of the nerve and exiting parallel to the transection site. Finally the suture was tied up to generate the loop-like shape of the nerve end. [file 12868_2017_374_MOESM1_ESM.tif]

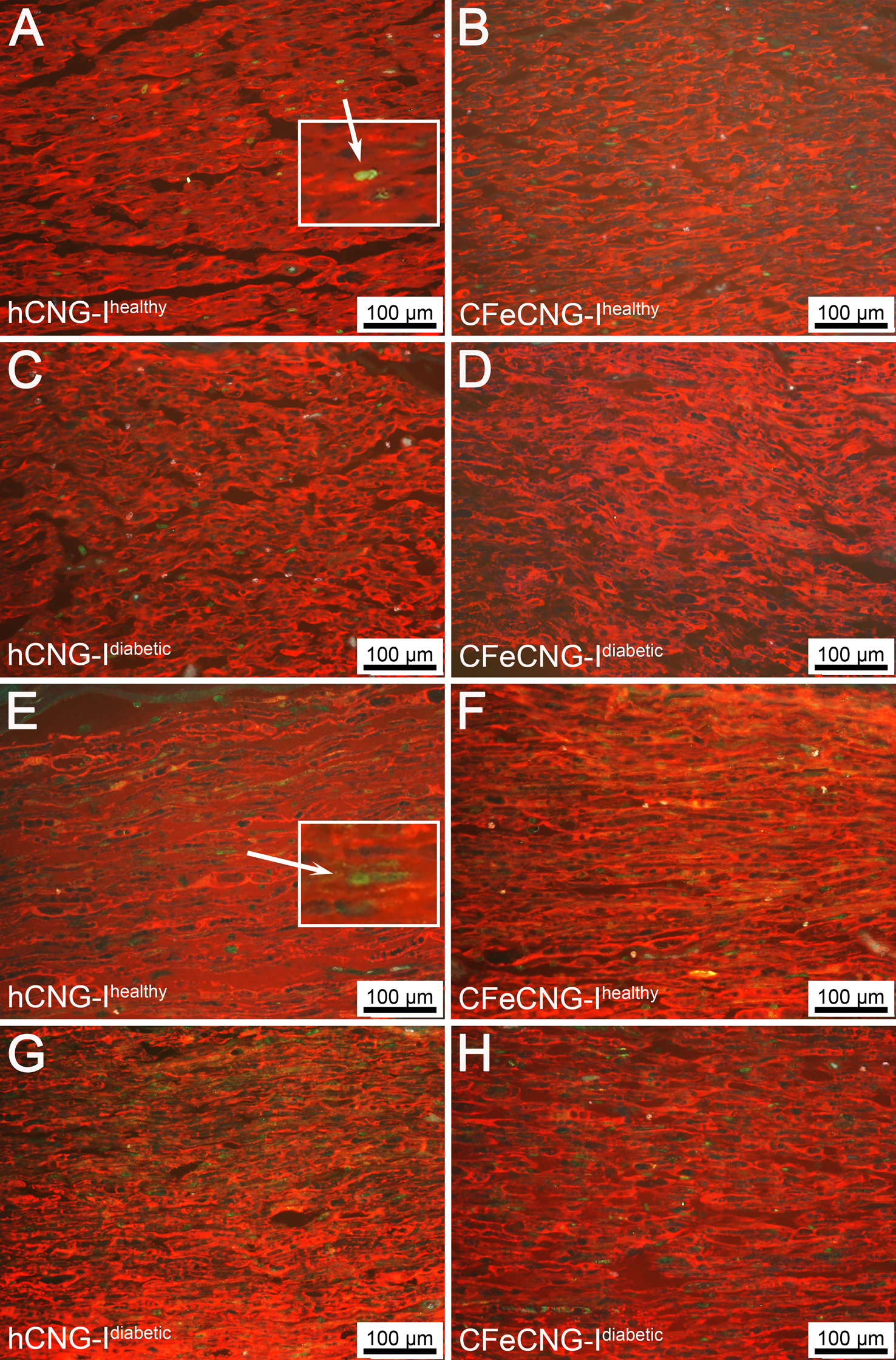

Supplement: Supplementary file 2 — Additional file 2: Figure S2. Detection of ATF-3 or cleaved caspase-3 stained Schwann cells in distal nerve segments after reconstruction. Photomicrographs present distal nerve segments at 56 days after 45 days delayed reconstruction double-stained for ATF-3 (A-D, in green) or cleaved caspase-3 (E-H, in green) with S-100 (A-H, in red). The inserts in A and E show a detail of a double-stained Schwann cell (S-100-immunopositive, red) with enclosed ATF-3 (green) or cleaved caspase-3 (green), respectively. Abbreviations: hCNG-Ihealthy = hollow chitosan nerve guide from healthy rats; CFeCNG-Ihealthy = chitosan film enhanced chitosan nerve guide from healthy rats; hCNG-Idiabetic = hollow chitosan nerve guide from diabetic GK rats; CFeCNG-Idiabetic = chitosan film enhanced chitosan nerve guide from diabetic rats. For detailed results of the quantification see Table 2. Scale bars display 100 µm in all images. [file 12868_2017_374_MOESM2_ESM.tif]

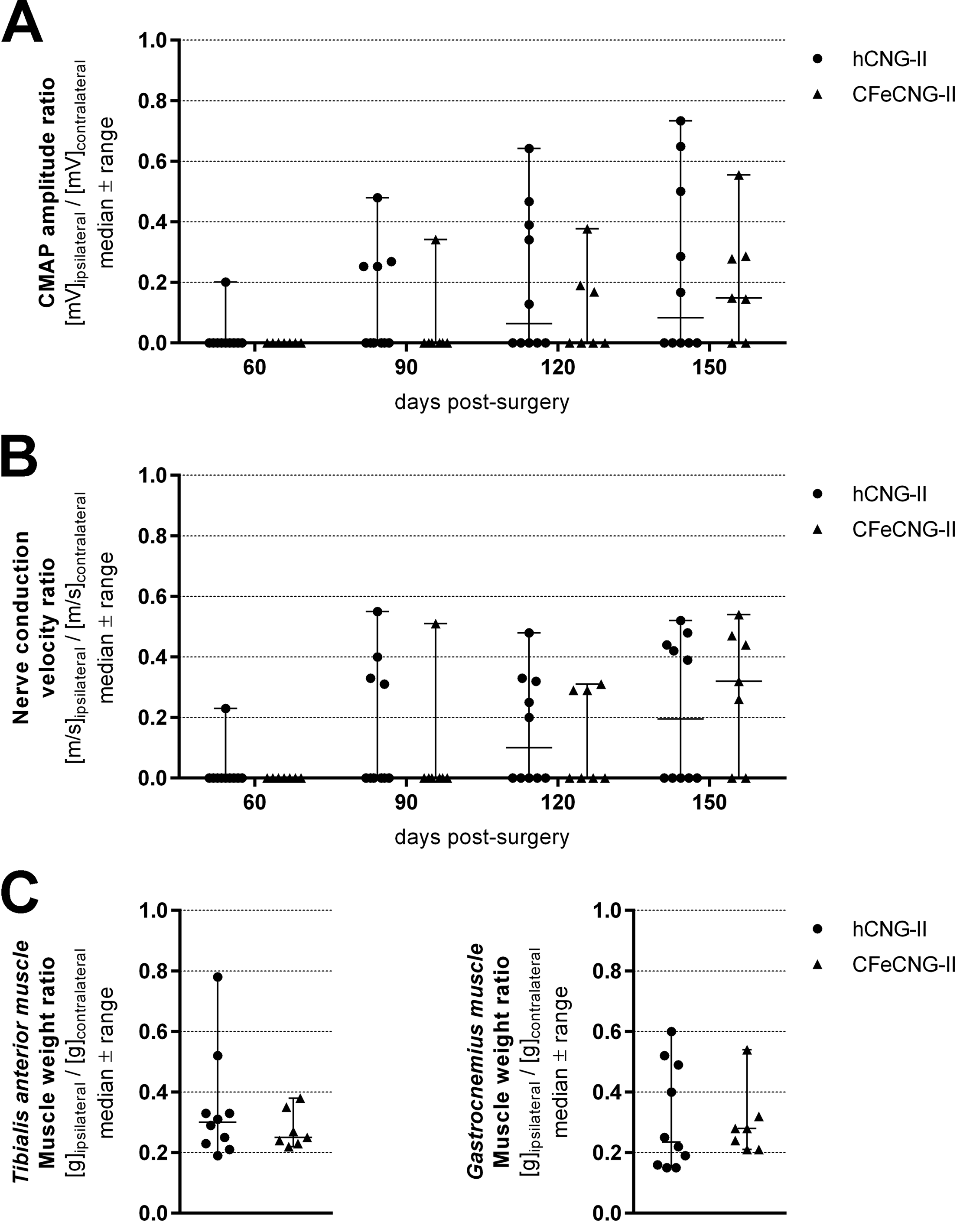

Supplement: Supplementary file 3 — Additional file 3: Figure S3. Quantitative analysis of motor recovery over 150 days post delayed reconstruction. As depicted by dot plots, the recovery of the evoked compound action muscle potential (CMAP) amplitude ratios (A) and the nerve conduction velocity ratios (B) displayed no significant quantitative difference between the two groups over the 150 day observation time after delayed nerve reconstruction. The lower limb muscle weight ratio (C) as determined at the endpoint of the study (150 days) did also not reveal any significant quantitative difference between the groups. Error bars indicate median ± range. [file 12868_2017_374_MOESM3_ESM.tif]

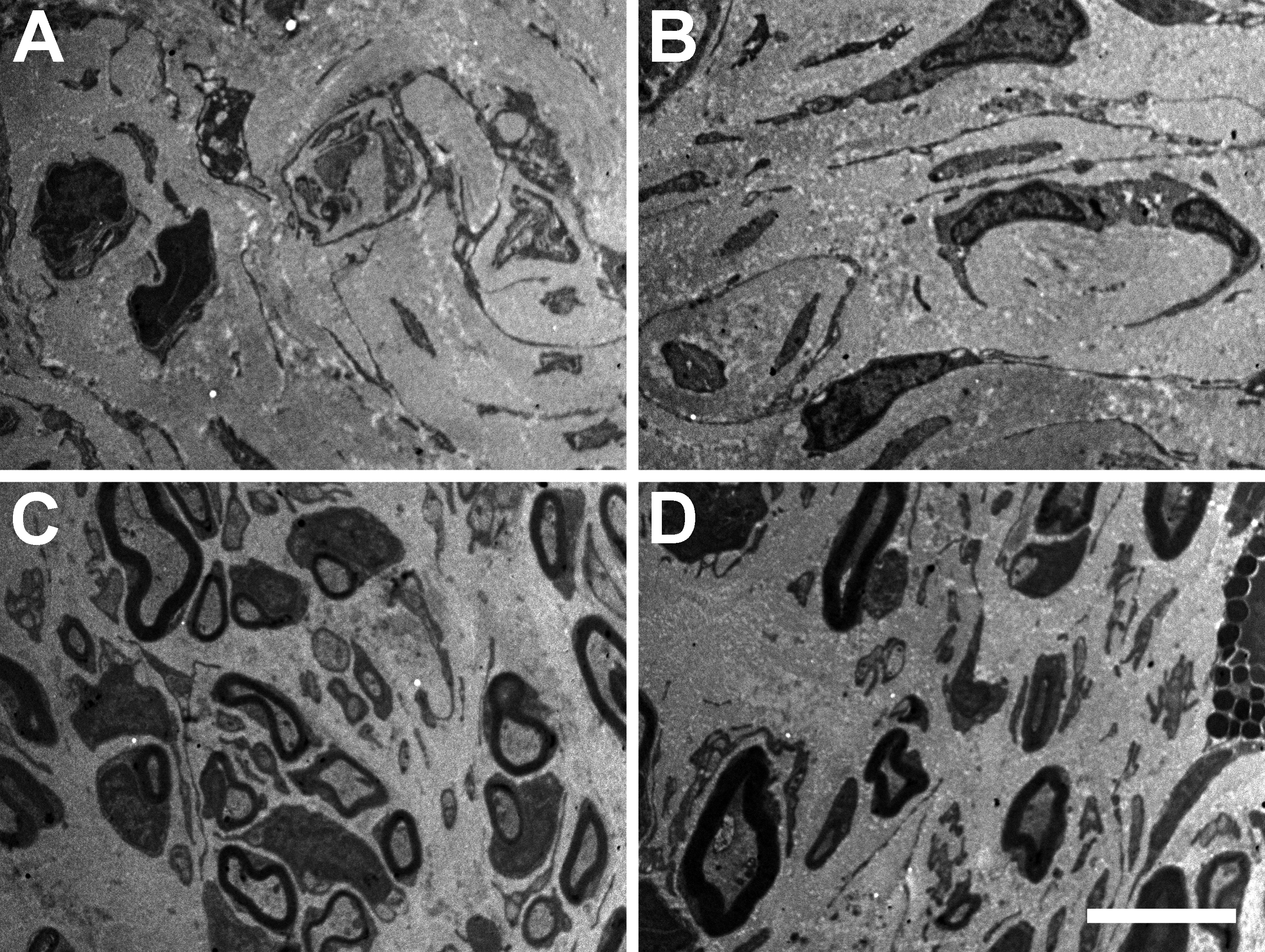

Supplement: Supplementary file 4 — Additional file 4: Figure S4. Representative high resolution images of ultrathin cross-sections. Photomicrographs show distal nerve segments of animals without an evocable CMAP, hCNG-II (A) and CFeCNG-II (B), in contrast to samples of animals with evocable CMAPs, hCNG-II (C) and CFeCNG-II (D), 150 days after reconstruction. Scale bar displays 5 µm for all pictures. [file 12868_2017_374_MOESM4_ESM.tif]
